# Characterising Mutational Spectra of Carcinogens in the Tumour Suppressor Gene *TP53* Using Human *TP53* Knock-in (Hupki) Mouse Embryo Fibroblasts

**DOI:** 10.3390/mps2040085

**Published:** 2019-11-13

**Authors:** Lisa Hölzl-Armstrong, Jill E. Kucab, Michael Korenjak, Mirjam Luijten, David H. Phillips, Jiri Zavadil, Volker M. Arlt

**Affiliations:** 1 Department of Analytical, Environmental and Forensic Sciences, King’s College London, London SE1 9NH, UK; lisa.hoelzl-armstrong@kcl.ac.uk (L.H.-A.); jill.kucab@kcl.ac.uk (J.E.K.); david.phillips@kcl.ac.uk (D.H.P.); 2 Molecular Mechanisms and Biomarkers Group, International Agency for Research on Cancer, 69008 Lyon, France; korenjakm@iarc.fr (M.K.); zavadilj@iarc.fr (J.Z.); 3 Center for Health Protection, National Institute for Public Health and the Environment (RIVM), 3721 MA Bilthoven, The Netherlands; mirjam.luijten@rivm.nl

**Keywords:** tumour suppressor p53, mutation, immortalisation, environmental carcinogenesis, genotoxicity, cancer aetiology, DNA adducts, Nutlin-3a, mouse model, embryonic fibroblasts

## Abstract

DNA in dividing cells is prone to mutagenesis, with mutations making key contributions to human disease including cancer. The tumour suppressor gene *TP53* is the most frequently mutated gene in human tumours. Here, we present a robust protocol for studying *TP53* mutagenesis utilising human *TP53* knock-in (Hupki) mouse embryonic fibroblasts (HUFs). In the HUF immortalisation assay (HIMA), primary HUFs are treated with known or suspected carcinogens at 3% oxygen and then transferred to 20% atmospheric oxygen to induce senescence. Cells containing mutations (e.g., in *TP53*) that allow bypassing of senescence eventually emerge as immortalised clonal cell lines after 2–3 months of serial passaging. As not all immortalised HUF cells contain *TP53* mutations, we developed a Nutlin-3a counter-screen to select for *TP53*-mutated clones prior to sequencing. *TP53* mutation spectra generated can be compared with those of human tumours recorded in the International Agency for Research on Cancer TP53 mutation database. Environmental mutagens that have demonstrated and validated the utility of the HIMA include ultraviolet radiation, aristolochic acid, and benzo[*a*]pyrene. The *TP53* mutation patterns induced by these mutagens in the HIMA corresponded to those found in human tumours from patients exposed to these mutagens. The approach presented helps to deepen our understanding of human cancer aetiology.

## 1. Introduction

The transcription factor p53 is usually kept at low levels in normal, unstressed cells, but it is stabilised and activated in response to certain stresses (e.g., DNA damage) [1]. This activation leads to a variety of outcomes such as cell cycle arrest, senescence or apoptosis depending on the severity of the damage. By preventing the growth of stressed and damaged cells, p53 plays a vital role in tumour suppression [2]. The function of p53 can be altered by mutations in the *TP53* gene that encodes for p53. *TP53* is the most commonly mutated gene in cancer with around 50% of all human tumours harbouring a mutation in *TP53*. These are mostly missense mutations occurring in the DNA binding domain encoded by exons 5–8 [3]. The International Agency for Research on Cancer (IARC) curates a database (www.p53.iarc.fr) of all mutations found in the *TP53* gene published in the scientific literature. This database currently lists around 30,000 mutations in human cancers. Most missense mutations in *TP53* cause a loss of function such that tumour suppressor capability is lost. However, some *TP53* mutations can lead to a gain of function, whereby the mutant p53 acquires a new activity [4].

**Figure 1:**
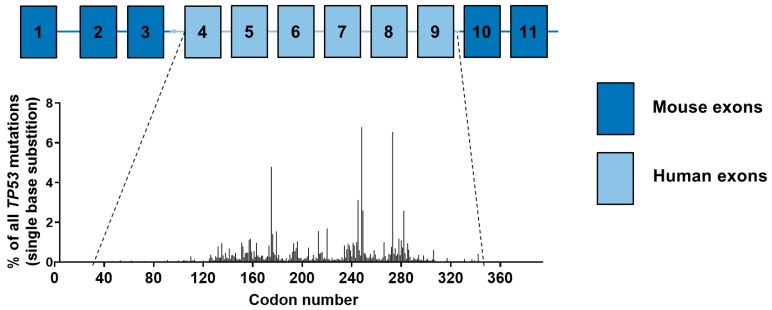
The *Hupki* mouse allele. Exons 4–9 of the mouse are replaced with the corresponding human exons. Most mutations in *TP53* of human tumours are found in these exons. Mutation data from human tumours were obtained from the IARC TP53 mutation database (www.p53.iarc.fr; R20 version).

A unique tool to study carcinogen-induced human *TP53* mutations in a mammalian cell context uses Hupki mouse embryo fibroblasts (HUFs) to perform the HUF immortalisation assay (HIMA). The Hupki mouse contains a partial human *TP53* knock-in allele, in which exons 4–9 of the murine *Trp53* gene have been replaced by the corresponding human exons, where most *TP53* mutations are found in human tumours (Figure 1) [5]. The p53 protein of the Hupki mouse functions normally and the mice are not cancer prone, unlike *Trp53* knockout mice which develop tumours (mostly lymphomas) at 3–6 months of age [5,6]. The key advantage of mouse embryo fibroblasts (MEFs) is that they undergo p53-dependent senescence after around 5–6 population doublings under normal culture conditions (37 °C, 20% O_2_, 5% CO_2_) [7,8]. MEFs can bypass senescence by a disruption of either the retinoblastoma or p53-protein pathway and thus, a mutation in *Trp53* is sufficient to immortalise MEFs. The immortalisation of human cells requires the disruption of both pathways in addition to a halt of telomere attrition [9]. It also takes much longer as human cells only enter senescence after 50–60 population doublings under standard culture conditions.

**Figure 2:**
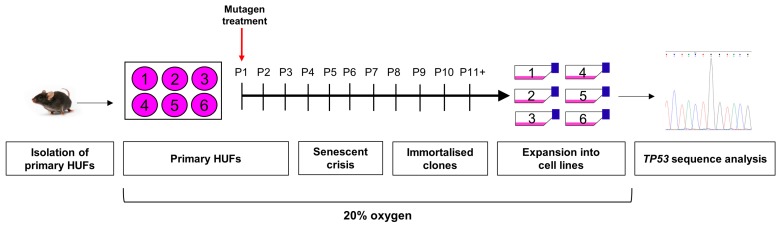
General experimental setup of the original HIMA [10]. Primary HUFs are isolated and grown under standard culture conditions (20% oxygen). For the HIMA primary HUFs are seeded on six-well plates (P1), treated with a mutagen and serially passaged under standard culture conditions (20% oxygen) until cells undergo senescence (P5+). Mutated cells eventually emerge from senescence and can be expanded into immortalised clonal cell lines (P8+). *TP53* sequencing with isolated DNA from all clones is performed to identify *TP53* mutations and to evaluate the pattern of mutations induced by the mutagen.

The original protocol for the HIMA was published by Liu et al. [10] (Figure 2). The assay is initiated by treating primary HUFs with a mutagen, followed by serial passaging of treated cells and untreated controls. Cultures will undergo growth arrest due to the sensitivity of MEFs to 20% oxygen. However, most mutagen-treated cultures will harbour mutated cells that are able to bypass senescence, start proliferating again and eventually become immortalised cell lines. Additionally, untreated cells can undergo spontaneous immortalisation due to mutations acquired through culture conditions (e.g., due to oxidative stress). DNA from immortalised cells can then be isolated and sequenced to identify *TP53* mutations [10] (Figure 2). Up to 30% of carcinogen-treated and 0–10% of untreated immortalised cultures harbour mutations in *TP53* [11,12,13,14,15], while the remaining immortalised cultures most likely have mutations in other genes related to senescence bypass [16]. The HIMA is a unique in vitro mutation assay as it assesses the mutagenesis of a human gene that plays an important role in cancer. Other in vitro mutation assays use either non-mammalian genes (e.g., *lacI*, *lacZ*, *cII*) or genes with no known role in cancer (e.g., *HPRT*, *TK*) [17,18,19,20].

## 2. Experimental Design

Prior to initiating the HIMA, mutagen treatment conditions must be optimised to ensure that sufficient DNA damage is induced while maintaining a population of viable cells. Therefore, the cytotoxicity of the known or suspected mutagen to be tested should first be assessed to identify a desirable concentration and an appropriate treatment time. It is important to note that the assessment of cytotoxicity helps to optimise treatment conditions for the HIMA, however, enhanced cytotoxicity is not necessarily a predictor of DNA damage and subsequent mutagenicity [21]. Thus, additional screening assays can help to further guide the HIMA treatment conditions.

When possible, DNA damage (e.g., pre-mutagenic DNA adducts) can be measured directly in mutagen treated cells (see Section 3.4.3). Alternatively, induction of the DNA damage response (DDR), after treatment with a variety of sub-cytotoxic and cytotoxic concentrations of a mutagen, can be assessed by western blotting or immunofluorescence for DDR proteins such as p53, p21, pChk1/2, and pH2ax (see Section 3.4.2). Additionally, we have created and isolated HUFs from the Hupki mouse with an integrated *lacZ* gene. The mutagenic activity of a known or suspected genotoxicant can be determined in *lacZ* and used as a reference point for identifying the level of mutations induced by the test agent in *lacZ* under different treatment conditions before starting the more laborious HIMA (see Section 3.4.4).

**Figure 3:**
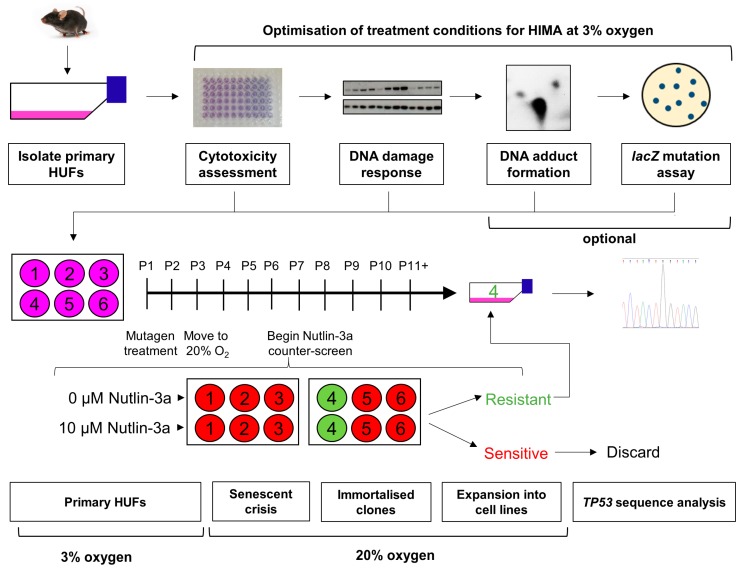
Updated experimental design for the HIMA. Primary HUFs are isolated and grown at 3% oxygen. Prior to the HIMA treatment conditions must be optimised. For this purpose, cells are treated with the mutagen of interest and cytotoxicity, DNA damage response, genotoxicity (i.e., DNA adduct formation) and/or *lacZ* mutagenicity are used to determine treatment conditions for the HIMA. For the HIMA primary HUFs are seeded on six-well plates (P1) and treated with the test agent using the optimised treatment conditions. Treatment is conducted at 3% oxygen. Cells are then serially passaged under standard culture conditions (20% oxygen) until they undergo senescence (P5+). Cells will eventually emerge from senescence and can be developed into immortalised clonal cell lines (P8+). The Nutlin-3a counter-screen is used to screen immortal clones for the presence of *TP53* mutations. Only *TP53*-mutated clones (i.e., Nutlin-3a resistant clones) will be expanded and subsequently subjected to *TP53* sequence analysis.

Over the past few years, we have developed and validated modifications to the original HIMA protocol to improve the sensitivity and applicability of the assay [13,15,22], and the updated experimental approach is illustrated in Figure 3. The two major changes are explained below.

First, we recommend culturing primary HUFs at 3% O_2_ instead of 20% O_2_ at the start of the assay (i.e., the first two weeks in culture) as well as when optimising treatment conditions. HUFs are highly sensitive to the oxidative stress that occurs when cultured under standard conditions (20% O_2_). Growth progressively slows until senescence occurs after approximately 2 weeks. HUFs grown at 3% O_2_ do not senesce and will undergo more population doublings than those grown at 20% O_2_, therefore culturing under lowered O_2_ produces more cells. Additionally, this may reduce the number of spontaneous, background mutations caused by oxidative damage to DNA. Following mutagen treatment, HUFs must then be transferred to 20% O_2_ to induce senescence and select for *TP53* mutations. We have shown that HUFs initially cultured at 3% oxygen that were transferred after 11 days to 20% oxygen were still able to senesce and become immortalised in the same timeframe as HUFs continuously cultured at 20% oxygen. Additionally, using this method, the percentage of spontaneous *TP53* mutations has been proven to be very low (0–4%).

Second, we recommend performing a counter-screen of immortalised HUFs with the MDM2 inhibitor Nutlin-3a. Nutlin-3a blocks the interaction between p53 and its negative regulator MDM2 by binding to the latter [23]. When cells with WT-*TP53* are treated with Nutlin-3a, p53 is stabilised and activated, leading to growth arrest [22]. Cells harbouring mutations in *TP53*, on the other hand, are resistant to Nutlin-3a and continue to grow normally after treatment. When following the original HIMA protocol (see Figure 2), all cultures are maintained from the first emergence of immortalised cells through the establishment of clonal cell lines, and all clones are sequenced for *TP53* mutations. The majority of these clones will not harbour a mutation in *TP53* but will have been immortalised by mutations in other genes. Therefore, because there is no selection specifically for *TP53* mutants in the original HIMA protocol, a lot of time and effort is spent culturing clones that have WT-*TP53*. Our group has demonstrated that a Nutlin-3a counter-screen, as shown in Figure 3, can be used to select for mutant-*TP53* HUFs and against WT-*TP53* HUFs once clones emerge from senescence [22]. Most mutant-*TP53* HUFs, including heterozygous *TP53* mutants that retain one WT allele, are identified by the Nutlin-3a counter-screen within 2.5 months of initiating the HIMA. Only *TP53*-mutant cultures are expanded into cell lines and *TP53*-WT cells can be discarded, improving the efficiency of the assay. This allows more cultures to be included in each experiment, more assays to be performed and thus more *TP53* mutants are processed [24].

The following Hupki mice are available to perform the described HIMA protocol:

- The original Hupki mouse (Trp53^tm1/Holl^) on a 129/Sv background encodes arginine at the exon 4 codon 72 polymorphic site in *TP53* [5]. More information can be found at The Jackson Laboratory website (www.jax.org) where this strain is available (stock no. 004301). The related strain Trp53^tm2/Holl^ (stock no. 008045) contains the proline variant at codon 72 in *TP53* [25]
- Based on the original Hupki mouse [5] we created a Hupki strain that is heterozygous for an *Xpa*-knockout allele (*Xpa^+/−^*) and also harbours the pUR288 plasmid (containing the bacterial *lacZ* reporter gene): Hupki/Xpa/lacZ [15]. The Hupki/Xpa/lacZ strain [B6;129-Trp53tm1holl-Xpatm1Hvs-Tg(pUR288)1Vij] is on a mixed 129/Sv and C57Bl/6 background. In the *Xpa*-knockout allele, exon 3, intron 3 and exon 4 have been replaced by a neomycin resistance cassette with a PGK2 promoter. By breeding *Hupki^+/+^*, *Xpa^+/−^* mice, *Xpa^+/+^* and *Xpa^−/−^* HUFs can be isolated. *Hupki^+/+^;Xpa^−/−^* cells are deficient in nucleotide excision repair. The pUR288 plasmid is chromosomally integrated in ∼20 tandem copies per haploid genome and allows mutagenesis of the *lacZ* gene to be assessed in HUFs (described in Section 3.4.4). More information can be found at the European Mouse Mutant Archive (EMMA; www.infrafrontier.eu) where this strain has been deposited (EMMA ID EM:08137).

### 2.1. Materials

#### 2.1.1. Mouse Dissection

- Basic dissecting set, i.e., sharp tweezers, blunt tweezers, fine scissors, blunt scissors (S Murray & Co Ltd.; Surrey, UK; Cat. no.: E251/01) or equivalent
- Dissecting microscope (Sigma-Aldrich; Cat. no.: Z738166)
- Dissection board and minimum of 8 needles for pinning (Thermo Fisher Scientific; Cat. no.: 13459386)

#### 2.1.2. Routine Cell Culture Reagents

- Dulbecco’s modified Eagle medium (DMEM)—high glucose (Thermo Fisher Scientific; Waltham, MA, USA; Cat. no.: 31966047)
- Penicillin/Streptomycin (P/S; Thermo Fisher Scientific; Cat. no.: 15140122)
- Foetal bovine serum (FBS; Thermo Fisher Scientific; Cat. no.: 10270106)
- Phosphate-buffered saline (PBS) pH 7.45 (Thermo Fisher Scientific; Cat. no.: 18912-014)
- Trypsin-EDTA 0.05% (Thermo Fisher Scientific; Cat. no.: 25300054)
- Ethanol 70% solution (Merck; Darmstadt, Germany; Cat. no.: 32221)
- Nutlin-3a (Cayman Chemicals; Ann Arbor, MI, USA; Cat. no.: 18585)
- Dimethyl sulfoxide (DMSO; Sigma Aldrich; St. Louis, MO, USA; Cat. no.: D2650)
- Culture-treated Nunc™ EasYFlasks™ in 25, 75 and 175 cm^2^ (Thermo Fisher Scientific; Cat. no.: 10568482, 10364131 and 159910)
- Corning^®^ CellBIND^®^ 6-well plates (Corning; New York, NY, USA; Cat. no.: 3335)
- Corning^®^ CellBIND^®^ 96-well plates (Corning; Cat. no.: 3330)
- Culture treated 6-well plates (Thermo Scientific; Cat. no.: D2650 10119831)
- Sterile 10-cm dishes (Corning, Cat. no.: 430591)
- Sterile serological pipettes 5, 10 and 25 mL (Corning; Cat. no.: 10127400, 10677341 and 10732742)
- Sterile centrifuge tubes 15 and 50 mL (Corning; Cat. no.: 10738771 and 10604551)
- Sterile 1.5 mL tubes (Corning; Cat. no.: 525-0259)
- Autoclaved glass Pasteur pipettes (Thermo Fisher Scientific; Cat. no.: 11566963)
- Sterile filter tips (Anachem; Leicester, UK; Cat. no.: GP20, GP200, GP1000)
- Cryogenic vials (Thermo Fisher Scientific; Cat. no.: 10577391)
- 12-well reservoir (VWR; Radnor, PA, USA; Cat. no.: 613-0100)

#### 2.1.3. DNA Isolation and PCR Reagents

- Gentra Puregene Cell Kit B (Qiagen; Hilden, Germany; Cat. no.: 158745)
- peqGOLD Cycle-Pure Kit (peqlab; Erlangen, Deutschland; Cat. no.: 12-6493-0)
- Isopropanol (Fisher Chemicals; Cat. no.: P17500/17)
- Ethanol 70% solution in nuclease-free water
- Nuclease-free water (Thermo Fisher Scientific; Cat. no.: AM9937)
- PCR Primers (Section 5)
- Tris-EDTA (TE; 10 mM Tris-HCl, 1 mM EDTA) pH 8.0 (Sigma Aldrich; Cat. no.: 93283)
- REDTaq^®^ ReadyMix™ (Sigma; Cat. no.: R2523-100RXN)
- UltraPure™ Agarose (Thermo Fisher Scientific; Cat. no.: 16500100)
- Ethidium bromide (Sigma-Aldrich; Cat. no.: E1510)
- DNA ladder 100 bp (Qiagen, Cat. no.: 239045)
- UltraPure™ 10X TBE buffer (Thermo Fisher Scientific; Cat. no.: 15581044)

#### 2.1.4. Reagents and Materials for Western Blotting

- EDTA (Sigma-Aldrich Cat. no.: EDS500G)
- Cling film
- Amersham ECL kit (GE Healthcare; Cat. no.: RPN2106)
- Amersham Hyperfilm ECL (GE Healthcare; Cat. no.: 28906836)
- Bromophenol blue salt (Sigma-Aldrich; Cat. no.: B0126)
- Filter paper
- Glycerol (Sigma-Aldrich; Cat. no.: G7757)
- Glycine (Santa Cruz; Cat. no.: sc-29096)
- Halt™ Protease-/Phosphatase Inhibitor Cocktail (100X) (Thermo Scientific; Cat. no.: 78430)
- Hydrochloric acid (VWR; Cat. no.: 20252-368)
- Loading tips (VWR; Cat. no.: 53509-015)
- Methanol (Merck; Cat. no.: 32213)
- Non-fat milk powder (Marvel)
- Sodium chloride (Sigma-Aldrich; Cat. no.: S3014)
- Nitrocellulose membrane (Bio-Rad, Hercules, CA, USA; Cat. no.: 162-0112)
- NuPAGE 4–12% Bis-Tris Protein Gels, 1.5 mm, 15-well (Invitrogen; Cat. no.: NP0336BOX)
- NuPAGE MES buffer (Invitrogen; Cat. no.: NP0002)
- Pierce Bicinchoninic Acid Protein Assay (Thermo Fisher Scientific; Cat. no.: 23225)
- Pierce Bovine Serum Albumin Standard Ampules (Thermo Fisher Scientific; Cat. no.: 23209)
- Ponceau red (Sigma-Aldrich; Cat. no.: P7170)
- SDS (AppliChem GmbH; Darmstadt, Germany; Cat. no.: A0767,0250)
- SeeBlue Plus2 Pre-stained Protein Ladder (Thermo Fisher Scientific; Cat. no.: LC5925)
- Sodium azide (Sigma-Aldrich; Cat. no.: S8032)
- Tris base (Thermo Fisher Scientific; Cat. no.: 17926)
- Tween-20 (GE Healthcare; Cat. no.: 28906838)
- β-Mercaptoethanol (Sigma-Aldrich; Cat. no.: M3148)
- Film cassette (Advansta; San Jose, CA, USA; Cat. no.: L-07019-001) or equivalent
- Scalpel (Swann-Morton; Sheffield, South Yorkshire, UK; Cat. no.: 12397999)
- Gel knife (Thermo Fisher Scientific; Cat. no.: EI9010)
- Roller (Thermo Fisher Scientific; Cat no.: 84747)

#### 2.1.5. Antibodies for Western Blotting

- anti-p53 (Cell Signaling Technology, Danvers, MA, USA; Cat. no.: 2524S)
- anti-p-p53 (Ser15; Cell Signaling Technology, Cat. no.: 9284)
- anti-p21 (BD Biosciences, Franklin Lakes, New Jersey, NJ, USA; Cat. no.: BD556431)
- anti-p-Chk1 (Ser345; Cell Signaling Technology; Cat. no.: 2348)
- anti-p-H2ax (Ser139; Cell Signaling Technology; Cat. no.: 9718
- anti-Mdm2 (abcam; Cambridge, Cambridgeshire, UK; Cat. no.: 28146)
- anti-Gapdh (Chemicon International, Temecula, CA, USA; Cat. no.: MAB374)
- anti-mouse (Bio-Rad; Cat. no.: 170-5046)
- anti-rabbit (Bio-Rad; Cat. no.: 170-5047)

### 2.2. Equipment

- Class 2 Biosafety cabinet (Walker; Glossop, UK; Cat. no.: Class II Gen3 Controls) or equivalent
- Incubator fitted with an oxygen sensor and a nitrogen source and capable of maintaining 37 °C, 5% CO_2_, 95% humidity and 3 or 21% O_2_ (Thermo Fisher Scientific; Cat. no.: Heracell™ 150i) or equivalent
- Culture microscope (Nikon, Minato, Tokyo, Japan; Cat. no.: Eclipse TS100) or equivalent
- Hemocytometer (Hawksley; Lancing, Sussex, UK; Cat. no.: BS.748) or equivalent
- Micropipettes (Gilson Scientific Ltd.; Middleton, WI, USA) or equivalent
- Multichannel pipette (Cole-Parmer; Vernon Hills, Il, USA; Cat. no.: P4808-200) or equivalent
- Multichannel aspirator adaptor (Thermo Fisher Scientific; Cat. no.: 10043471) or equivalent
- Pipetboy (Integra Biosciences; Zizers, Switzerland; Cat. no.: 15016)
- Centrifuge: 320× *g* and 2300× *g* (Eppendorf; Hamburg, Germany; Cat. no.: 5415D and 5804) or equivalent
- NanoDrop microvolume spectrophotometer (Thermo Fisher Scientific; Cat. no.: ND-2000) or equivalent
- Thermocycler (Eppendorf; Cat. no.: Thermomixer compact 5350) or equivalent
- Vortex (Scientific Industries; Bohemia, NY, USA; SI-0236) or equivalent
- Gel electrophoresis tank (Gibco; Cat. no.: 11068) or equivalent or equivalent
- Sonic dismembrator (Fisher Scientific; Cat. no.: FB-120) or equivalent
- Microplate reader for absorbance (BioTek; Winooski, VT, USA; Cat. no.: ELx800) with Gen5 software (BioTek) or equivalent
- PowerPac (Bio-Rad; Cat. no.: 1645052) or equivalent
- XCell SureLock Mini-Cell Electrophoresis System (Thermo Fisher Scientific; Cat. no.: EI0001) or equivalent
- Mini Trans-Blot Electrophoretic Transfer Cell (Bio-Rad; Cat. no.: 1703930) or equivalent
- pH meter (Hanna Instruments; Leighton Buzzards, Bedfordshire, UK; Cat. no.: HI220) or equivalent
- Medical film processor (Konica Minolta; Chiyoda, Tokyo, Japan; Cat. no.: SRX-101A) or equivalent

## 3. Procedure

### 3.1. Isolation of Primary Mouse Embryo Fibroblasts (Time for Completion: 18 Days)

#### 3.1.1. Mouse Work and Dissection

1. For mating place one male with 1–2 females (7–8-week virgins) and check the females for a copulation plug each morning. The copulation plug usually dissolves within 12–14 h.
2. Transfer the plugged females to a new cage (=E0.5) and weigh the female on day 7, 9, 11, and 13.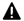 **CRITICAL STEP** The optimal age of mouse embryos for this protocol is 13.5 days.
3. Prior to dissection (E13.5) place medium at room temperature. Place 8–10 cryogenic vials containing 1 mL 0.05% trypsin-EDTA on ice. Prepare 8–10 10-cm petri dishes with approximately 20 mL sterile PBS. Put dissection tools in a beaker containing sterile PBS.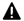 **CRITICAL STEP** The isolation of MEFs must occur in a sterile hood with sterile material.
4. Sacrifice the female by cervical dislocation.
5. Spray the mouse on both sides with 70% ethanol and pin down.
6. With fine tweezers or scissors, carefully cut open the mouse and pin down the skin on both sides.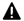 **CRITICAL STEP** Do not touch fur with dissection tools in order to avoid contamination.
7. Cut out the string of embryos/uterus and place in 10-cm dish containing PBS.
8. Pop out each embryo from the uterus and place each in a separate 10-cm dish containing PBS.
9. Label 1.5 mL centrifuge tubes and cryogenic vials containing trypsin (from step 3) according to number of embryos and place back on ice.
10. Under the microscope remove the placenta and umbilical cord if still present.
11. Hold the embryo with sharp tweezers and remove the hematopoietic tissue with blunt tweezers.
12. Cut off the head with blunt scissors and place in a labelled centrifuge tube from step 9 and use for genotyping if necessary.
13. Place the rest of the embryo in a labelled trypsin-containing cryogenic vial from step 9 and keep on ice.
14. In between each embryo, wash dissection tools in a beaker containing PBS, then with 70% ethanol and dab on tissue to dry.

#### 3.1.2. Preparation of Cells

**NOTE** The day of HUF isolation is defined as Day 0 (or D0) of the cells being in culture.

15. Prepare HUFs from up to four embryos at a time.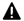 **CRITICAL STEP** From here onwards the preparation of HUFs must occur in a sterile cell culture biosafety cabinet.
16. Between dissociation of each embryo, rinse mincing scissors in a 50 mL centrifuge tube filled with PBS, then soak with 70% ethanol and dab on tissue to dry.
17. Use mincing scissors to mince the embryo in each vial.
18. Put up to four vials in a rack and place in the incubator at 37 °C for 10 min.
19. Pipette the embryo/trypsin mixture up and down 10× with 1 mL pipette.
20. Place the rack back in the incubator at 37 °C for a further 10 min.
21. Pipette the tissue up and down 10× with a 1 mL pipette.
22. Transfer the contents of each vial to separate 15 mL centrifuge tubes containing 9 mL of growth medium and invert. Centrifuge at 320× *g* for 5 min.
23. Aspirate supernatant and re-suspend the pellet in 1 mL medium by pipetting up and down 10×.
24. Add 4 mL of medium (total of 5 mL), mix, then let the tube sit for 3 min to sediment any large pieces of tissue.
25. Transfer 5 mL (avoiding pellet) from each tube to a separate 175-cm^2^ flask containing 30 mL medium.
26. Move the flask back and forth and place in incubator.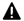 **CRITICAL STEP** The incubator should be fitted with an oxygen sensor and a nitrogen source and set to 37 °C, 95% humidity, 5% CO_2_, and 3% O_2_.
27. Check cell growth and for any signs of contamination over the next 2–3 days.
28. Change the medium after 24 h.

#### 3.1.3. Preparation of Frozen Cell Stocks

29. Label one 15 mL tube per T175 flask with the appropriate embryo number.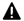 **CRITICAL STEP** It is possible to prepare frozen stocks from up to four flasks at a time. Because the freezing medium contains a high percentage of DMSO, it is important that cells are placed at −80 °C immediately to avoid any cellular damage.
30. For each embryo/flask label 5–6 cryogenic vials with ID, day in culture, passage number, portion of flask per stock and date (e.g., XE7.4, D3, P0, ⅕ T175, 01/01/2019).
  **NOTE** As described in Section 3.1.2 the day of isolation is defined as Day 0. In our experience, when following this procedure, most cultures will be 80–100% confluent three days following isolation, thus, the day when frozen stocks are prepared will be Day 3 (D3). Also, we define the preparation of frozen stocks here as Passage 0 (P0).
31. Aspirate the medium from the flask and wash with PBS.
32. Incubate with trypsin for 3 min, then add medium as indicated in Table 1, transfer to 15 mL centrifuge tube and centrifuge at 320× *g* for 5 min at room temperature.
33. Prepare freezing medium.
34. Aspirate the supernatant and re-suspend the pellet in 5 mL freezing medium by gently pipetting up and down.
35. Add 1 mL into each of the prepared cryogenic vials.
36. Place in a Styrofoam box at −80 °C and transfer to a liquid nitrogen tank the next day.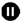 **PAUSE STEP** Frozen stocks can be stored in liquid nitrogen for many years.

### 3.2. Thawing of Frozen Cells. Time for Completion: 30 min Hands-on Time, 3–4 Days for Cells to Grow (D3–6/7)

37. Label two T175 flasks with embryo number, day in culture, passage number and date (i.e., XE7.4, D3, P0) and add 35 mL of medium to each flask.
38. Prepare one 15 mL tube with 9 mL medium.
39. Wearing the appropriate protective clothes, take out a vial of frozen primary cells from the liquid nitrogen tank and thaw quickly in a 37 °C water bath.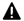 **CRITICAL STEP** As cells were frozen in 10% DMSO it is crucial to work fast at this step to keep cells viable.
40. In the cell culture hood, pipette the thawed stock into the 15 mL tube from step 38 and centrifuge at 280× *g* for 5 min at room temperature.
41. Aspirate the supernatant, resuspend the pellet in 1 mL of medium and add 500 µL of cell suspension per flask.
42. To ensure an even distribution of the cells, move the flasks back and forth before placing them in an incubator set to 37 °C, 95% humidity, 5% CO_2_, and 3% O_2_.
43. The next day (D4) aspirate the medium, add 35 mL fresh medium to each flask and place flasks back into the incubator.
44. Let the cells grow for an additional 48–72 h in order to reach 80–90% confluency.

### 3.3. Passaging of HUFs—Time for Completion: 30–45 min for Primary Cells and up to 4 h during the HIMA

45. Once cells have reached confluency (D6–7) label the required number of cell culture dishes with culture ID, date, day and passage number and add appropriate volume of growth medium as indicated in Table 1.
46. Take flasks out of the incubator and aspirate the medium. Wash once with 12 mL PBS per flask. Add 3 mL of trypsin. Make sure the trypsin has been distributed equally and place flasks at 37 °C for 2.5 min or until cells detach.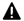 **CRITICAL STEP** Timing depends on the cell density and the stage of the culture (e.g., senescent cells take longer to dissociate than post-senescent cells or primary HUFs). Do not incubate cells longer than 10 min.
47. Check under the microscope if cells have detached. If cells are still attached place back at 37 °C. Once cells have lifted, add growth medium to inactivate trypsin and transfer the cell suspension to a 50 mL centrifuge tube.
48. Count cells using a hemocytometer and seed cells at 16,000 cells/cm^2^.
49. During the HIMA, after cells have been treated with a mutagen, the cells do not have to be counted. The splitting ratio depends on how quickly the culture populates the well. Cultures should be sub-cultured at 1:1.5–1:50 splitting ratio as indicated in Table 2. For example, for a 1:3 splitting ratio pipette 500 μL of the 1.5-mL cell suspension into the correct well of the prepared 6-well plate.
50. To ensure an even distribution of the cells, move the flasks/plates back and forth before placing them in the incubator at 37 °C, 95% humidity, 5% CO_2_, and 3% (D0–D11) or 20% O_2_ (D11+).

### 3.4. Optimisation of Carcinogen Treatment Conditions for HIMA

#### 3.4.1. Assessment of Cell Survival

##### Carcinogen Treatment of Cells—Time for Completion: 30 Min/Compound Followed by Incubation

**Figure 4:**
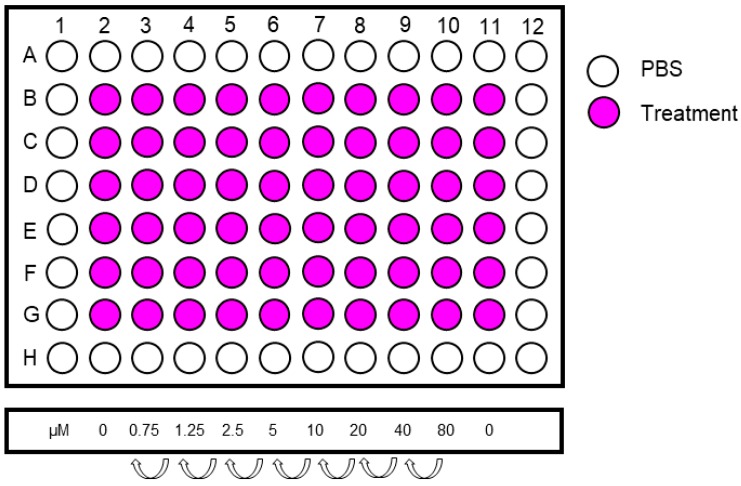
Example of 96-well plate set-up for cytotoxicity assessment. Columns 2 and 11 represent the controls, while columns 3–9 show various treatment concentrations. The outside wells are filled with PBS.

51. Prepare required amount of 96-well plates by labelling them with the date and passage number and by adding 100 μL PBS/well into the outside wells of the plate using a multichannel pipette.
52. Sub-culture the cells as described in Section 3.3 and place them in the incubator at 37 °C, 95% humidity, 5% CO_2_ and 3% O_2_. Prepare enough cells for 70 wells.
53. The next day prepare sufficient amounts of the highest concentration of the test compound by adding the required volume of carcinogen stock to medium. Also make medium containing an equal concentration of the solvent for the controls.
54. Make a serial dilution from the highest concentration in a 12-well reservoir. Include 6 replicates per treatment concentration. An example of treatment concentrations is shown in Figure 4.
55. Include two columns (=12 replicates) of controls.
56. Aspirate the medium from the cells using a multichannel aspirator adaptor or a multichannel pipette and dispose of accordingly.
57. Add 100 μL/well of treatment medium using a multichannel pipette and incubate for appropriate time (see Section 4.1 for advice).

##### Staining of Cells with Crystal Violet—Time for Completion: 30 min Followed by Drying Step (at least 1 h) and then Another 15 min the Next Day

**Figure 5:**
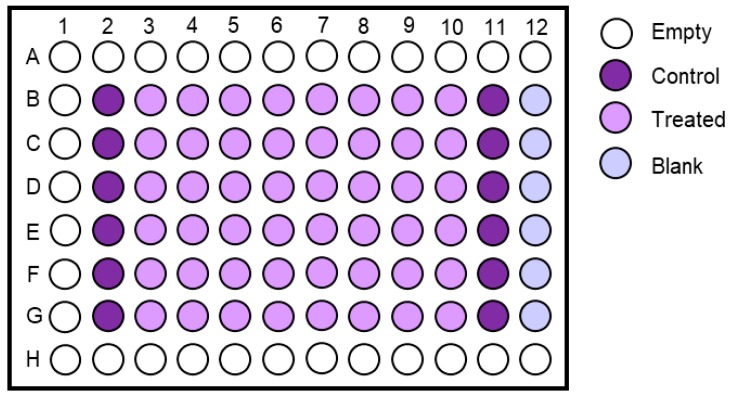
Example of 96-well plate during crystal violet staining. Columns 2 and 11 represent the controls (darker, as there are more cells), while columns 3–9 show various treatment concentrations (lighter, as there are less cells). Column 12 represents the blank wells, which contain no cells but are stained with Crystal violet.

58. Remove the treatment medium and PBS from plates using a multichannel pipette.
59. Wash cells with 150 μL PBS/well and subsequently remove PBS with multichannel pipette.
60. Add 35 µL of 0.1% crystal violet in 10% ethanol to each well, as well as one row without cells (=blank).
61. Incubate at room temperature for at least 10 min.
62. Remove the crystal violet solution using a multichannel pipette and dispose of accordingly.
63. Wash 2× with 150 μL PBS.
64. Let the plate dry for at least 1 h shielded from light.
65. When dry add 100 μL of 50% ethanol to each well, tap to mix, and measure absorbance at 595 nm using a plate reader (Figure 5).

##### Calculation of Cell Survival—Time for Completion: 15 Min

66. Calculate the mean blank absorbance and subtract it from each absorbance value.
67. Calculate the mean control absorbance value.
68. Calculate the cell survival as % control using the following formula:
  $$\mathrm{Cell}\;\mathrm{survival}\;\left(\%\;\mathrm{control}\right)=\frac{\;\left[\mathrm{absorbance}\;\mathrm{of}\;\mathrm{treated}\;\mathrm{cells}\;\left(\mathrm{nm}\right)-\mathrm{absorbance}\;\mathrm{of}\;\mathrm{blank}\;\left(\mathrm{nm}\right)\right]\text{×}100\%}{\left[\mathrm{absorbance}\;\mathrm{of}\;\mathrm{control}\;\mathrm{cells}\;\left(\mathrm{nm}\right)-\mathrm{absorbance}\;\mathrm{of}\;\mathrm{blank}\;\left(\mathrm{nm}\right)\right]}$$
69. Calculate the mean and standard deviation of each treatment condition. The standard deviation should be below 15%.

#### 3.4.2. Assessment of DDR Following Carcinogen Treatment

##### Carcinogen Treatment of Cells—Time for Completion: Set-Up of Experiment: 30 Min Followed by Overnight Incubation, Treatment: 30 Min/Compound

70. Prepare required amount of 6-well plates by labelling them with the date and passage number.
71. Sub-culture the cells as described in Section 3.3 and place them in the incubator at 37 °C, 95% humidity, 5% CO_2_, and 3% O_2_. Prepare at least four wells/compound (1 control and 3 different treatment concentration, see Section 4.1 for further advice).
72. The next day prepare sufficient amounts of the highest concentration of the test compound by adding the required volume of carcinogen stock to medium. Also make medium containing an equal concentration of the solvent for the controls.
73. Make a serial dilution from the highest concentration in tubes.
74. Aspirate the medium and dispose of accordingly.
75. Add 2 mL/well of treatment medium and place cells back into incubator for appropriate time (see Section 4.1).

##### Preparation of Cell Lysates—Time for Completion: 15 Min

76. When treatment time is finished, label microcentrifuge tubes with treatment conditions.
77. Prepare required amount of lysis buffer with protease/phosphatase inhibitors (Table 3—Section 5).
78. Wash cells once with PBS and add 80–130 μL lysis buffer/well.
79. Collect lysates in the corner of the dish.
80. Pipette lysates into labelled microcentrifuge tubes from step 76 and store at −20 °C.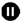 **PAUSE STEP** Cell lysates can be stored at −20 °C for several months until analysis.

##### Protein Quantification and Normalisation—Time for Completion: 90 Min

81. Before assaying protein concentration, sonicate lysates twice at 20% amp, 0 pulse, 10 sec to shear genomic DNA.
82. To set up a standard curve, add 5 µL of albumin standard dilutions (Table 4—Section 5) and 5 µL of lysis buffer (blank) in duplicate on a 96-well plate. Then add 5 µL of each lysate in duplicate on the 96-well plate.
83. Prepare working reagent by mixing 50 parts Reagent A and 1 part Reagent B from the Pierce Bicinchoninic Acid (BCA) Protein Assay.
84. Add 195 µL working reagent to each well with a multichannel pipette.
85. Mix the plate by gently tapping on the bench.
86. Incubate at 37 °C for 20 min.
87. Read absorption with a plate reader at 595 nm.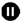 **PAUSE STEP** Samples can be stored at −20 °C or continue with step 88.
88. Calculate protein concentration and normalise samples to the lowest protein concentration with appropriate volumes of lysis buffer. Add bromophenol-blue and 1% β-mercaptoethanol to prepare samples for SDS-PAGE.
89. Heat samples at 95 °C for 5 min to denature proteins.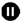 **PAUSE STEP** Samples can be stored at −20 °C or continue with step 90.

##### SDS-Polyacrylamide Gel Electrophoresis (SDS-PAGE)—Time for Completion: 2 h

90. Obtain a precast gel and remove the white tape and comb.
91. Assemble gel in gel tank and add running buffer.
92. Rinse the wells of the gel by pipetting fresh running buffer up and down.
93. Load 5 µL of protein marker in the leftmost well with a gel loading tip.
94. Load samples with gel loading tips accordingly (max. volume 21 μL).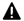 **CRITICAL STEP** Group samples starting with control up to highest concentration. Load a minimum of 15 µg protein.
95. Press down lid on chamber, attach cables and run at 150 V for 1.5 h (1.25 h if you include pH2ax).

##### Transfer—Time for Completion: 2 h

96. Cut nitrocellulose membrane and filter paper at 8 × 7 cm.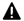 **CRITICAL STEP** Avoid touching the membrane with fingers or gloves. Only touch on the protective paper.
97. Pour transfer buffer into a plastic container.
98. Soak two sponges in the buffer and press out air bubbles with a roller.
99. Disassemble gel cassette by opening the plastic casing using a spatula on all sides.
100. Remove the top and bottom of the gel.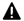 **CRITICAL STEP** As the gel is very fragile care should be taken to avoid breakage.
101. Label pre-cut nitrocellulose membrane with the date and gel number with a pencil.
102. Assemble ‘sandwich’: black (negative) part of blotting cassette—sponge—soaked filter paper—gel—nitrocellulose membrane—soaked filter paper—sponge—red (positive) part of cassette.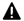 **CRITICAL STEP** Roll out air bubbles with a roller as they interfere with protein transfer.
103. Close the sandwich cassette and lock in chamber.
104. Add transfer buffer in blotting tank up to line ‘blotting’.
105. Place cool pack and stir bar inside the tank and set on magnetic stirplate.
106. Press down the lid on the tank, attach cables and transfer at 110 V for 1.5 h.
107. Change cool pack after 45 min.

##### Blocking. Time for Completion: 90 Min

108. Disassemble the sandwich cassette.
109. Stain the blot with Ponceau red in a box to assess protein transfer.
110. Rinse the blot in another box with water to remove background Ponceau stain.
111. Cut the blot at 28 kDa using a ruler and a scalpel on a plastic sheet.
112. Place the blot in another box with TBST to remove all Ponceau stain.
113. Block the membrane in 3% milk in TBST on a shaker at room temperature for 1 h.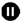 **PAUSE STEP** Blots can be stored in TBST at 4 °C for up to 3 days.

##### Primary Antibody. Time for Completion: Overnight

114. Discard blocking solution.
115. Transfer top part of membrane (>28 kDa) into p-p53/pChk1-antibody solution and lower part (<28 kDa) into p21/pH2ax-antibody solution with protein side facing upwards (Table 5—Section 5).
116. Incubate blots overnight at 4 °C on a rotator.

##### Secondary Antibody. Time for Completion: 90 Min

117. Place blots in separate boxes with TBST on a shaker and primary antibody solution back at −20 °C.
118. Wash blots three times for 10 min with TBST.
119. Prepare a secondary antibody solution during the last wash (Table 6—Section 5).
120. Place blots into the secondary antibody solution (protein facing upwards).
121. Incubate on a shaker at room temperature for 1 h.

##### Detection of Protein Bands. Time for Completion: 30 Min

122. Discard secondary antibody solution.
123. Wash blots twice for 10 min, followed by 20 min on a shaker at room temperature with TBST.
124. Arrange membranes on a piece of cling film (protein facing up, ladder on the left).
125. Prepare an appropriate volume of enhanced chemiluminescence (ECL) solution by mixing equal parts of Reagent A and Reagent B of the Amersham ECL kit.
126. Pipette 3 mL of the ECL solution onto each membrane.
127. Incubate membranes with ECL solution for 1 min.
128. Close cling film around the membranes and remove all excess liquid with a paper towel.
129. Secure the membranes in a film cassette by taping down the edges of the cling film.
130. Expose to film (exposure time ~1–5 min) and then process the film using a medical film processor.

##### Reprobing a Western Blot for Loading Control (Gapdh)—Time for Completion: 3.5 h

131. Continuing from step 130, remove blots from cling film and wash for 10 min in TBST.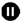 **PAUSE STEP** Blots can be stored in TBST at 4 °C for up to 3 days.
132. Place top blot (>28 kDa) into Gapdh-antibody solution and incubate for 30 min at room temperature on a shaker.
133. Repeat steps 117–130 and store blots at −20 °C.

#### 3.4.3. Assessment of Pre-Mutagenic DNA Adducts (Optional)

134. Thaw appropriate number of vials of primary HUFs as described in Section 3.2. 3–4 days prior to setting up an experiment for the DNA adduct analysis.
135. Once cells have reached confluency, label the appropriate number of 75-cm^2^ flasks.
136. Trypsinise and count the cells as described in Section 3.3 and seed 1.2 × 10^6^ cells per flask.
137. Place the flasks in the incubator at 37 °C, 95% humidity, 5% CO_2_, and 3% O_2_.
138. The next day treat the cells with test compound and place flask back in the incubator.
139. Once the treatment is finished, prepare a pellet as described in Section 3.6.
140. DNA adducts can be analysed by a variety of different methods [26]. In our laboratory we routinely use the ^32^P-postlabelling assay to evaluate DNA adduct formation. We have published detailed step-by-step protocols previously [27,28].

#### 3.4.4. Assessment of lacZ Mutagenicity (Optional)

141. Thaw appropriate number of vials of primary HUFs as described in Section 3.2 3–4 days prior to setting up an experiment to assess the *lacZ* mutagenicity.
142. Once cells have reached confluency, label the appropriate number of 75-cm^2^ flasks (five per treatment condition).
143. Trypsinise and count the cells as described in Section 3.3 and seed 1.2 × 10^6^ cells per flask.
144. Place the flasks in the incubator at 37 °C, 95% humidity, 5% CO_2_ and 3% O_2_.
145. The next day treat the cells with test compound and place flasks back in the incubator.
146. Once the treatment is finished, label the required number of 175-cm^2^ flasks.
147. Trypsinise and count the cells as described in Section 3.3 and seed 2 × 10^6^ cells from each 75-cm^2^ flask into a 175-cm^2^ flask.
148. After three days, prepare a pellet as described in Section 3.6.
149. To evaluate the frequency of *lacZ* mutants in *E. coli* follow the protocol previously described [29].

### 3.5. Conducting the HIMA

#### 3.5.1. Seeding Cells for the HIMA. Time for Completion: 45 Min

150. Thaw appropriate number of vials of primary HUFs as described in Section 3.2 3–4 days prior to conducting the HIMA.
151. Once primary HUFs have reached confluency (D6–7), label desired amount of Corning^®^ CellBIND^®^ 6-well plates with date, day and passage number (D6, P1). For simplicity, this protocol defines the plating day as D6. Assign each well a culture ID. Make sure to differ between culture ID of treated and untreated cultures (e.g., X-1 for treated; C-1 for control).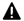 **CRITICAL STEP** Each HIMA should consist of at least 48 treated cultures; the upper limit depends on the experience of the scientist. Additionally, each HIMA should include an appropriate number of controls. Initially, we recommend at least 36 controls, but fewer controls (e.g., 6–12) can be included in subsequent assays when performing consecutive HIMAs in the same laboratory and by the same operator.
152. Detach the cells as described in Section 3.3.
153. Count cells using a hemocytometer and seed cells at 150,000 cells/well in 2 mL medium per well into 6-well plates.
154. To ensure an even distribution of the cells, move the flasks back and forth before placing them in the incubator at 37 °C, 95% humidity, 5% CO_2_, and 3% O_2_.

#### 3.5.2. Carcinogen Treatment of HUFs and Pre-Senescence Culture—Time for Completion: 30 Min for Treatment, Followed by at Least 11 Days (D7–18)

155. The following day (D7) prepare treatment medium at the desired concentration.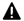 **CRITICAL STEP** Select the desired concentration for treatment based on the assessments conducted in Section 3.4 as discussed in Section 4.1.
156. Aspirate growth medium and add treatment medium. Incubate with the treatment medium for the desired time.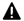 **CRITICAL STEP** Select the desired incubation time for treatment based on the assessments conducted in Section 3.4 as discussed in Section 4.1.
157. After the treatment period (D7–9), check cultures under the microscope and remove the treatment medium. Dispose of treatment medium appropriately in hazardous waste. Add 2 mL of fresh medium per well and place cells back into the incubator.
158. **NOTE** The confluency of the cells is dependent on the carcinogen concentration as well as treatment time tested which both can impact on cytotoxicity. The control wells are confluent 48 h post-treatment (D9). At this stage controls grow faster than treated cultures. It has been noticed that dependent on the carcinogen tested, growth of treated cells can slow down significantly.
159. Check cultures daily under a microscope and passage at ratios indicated in Table 2 and as described in Section 3.3 once a culture has reached 80–90% confluency.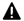 **CRITICAL STEP** As each well is a separate culture, evaluate its confluency separately. It is normal that some cultures grow faster or slower than other cultures and enter or exit senescence at a different pace. Dip the Pasteur pipette into 70% ethanol between aspirating media from different cultures to avoid carry-over of one culture to another.
160. Move cells to an incubator set to 37 °C, 95% humidity, 5% CO_2_, and 20% O_2_ five days post-treatment (D11) to induce senescence crisis.

#### 3.5.3. Culture of HUFs during Senescence Crisis—Time for Completion: At Least 8 Days

161. Continue checking the cells at least every second day under microscope.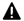 **CRITICAL STEP** During senescence the morphology of the cells will change. Senescent cells look elongated, flat and they stop dividing.
162. Change the medium every three days.
163. **NOTE** During senescence it is normal that splitting a culture is often not required for several days. However, if a culture has not continued growth after 14 days, passage all cells (1:1) to a new well.

#### 3.5.4. Culture of HUFs Post-Senescence and Nutlin-3a Counter-Screen—Time for Completion: ~2 Months (D32–90)

**Figure 6:**
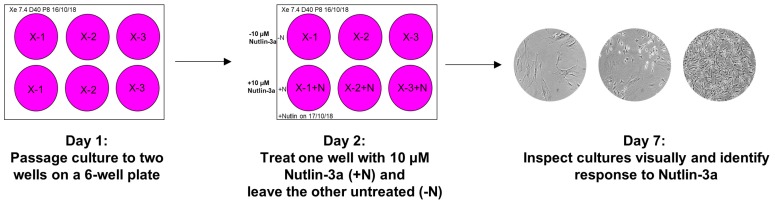
Labelling of 6-well plates for Nutlin-3a counter-screen.

164. Continue checking the cells at least every 2–3 days under the microscope until first clones are emerging.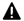 **CRITICAL STEP** When the first clones are emerging, wait until they have populated most of the well (at least half) rather than a smaller fraction of it. It is always recommended to split cells at lower dilutions initially (e.g., 1:1.5) and gradually go higher once the culture can repopulate the well quickly (e.g., 1:4, then 1:10). It is better to split the culture the next day again rather than losing the culture due to too harsh splitting. Cells can now be cultured in standard 6-well plates.
  **NOTE** Individual clones visually appear different: they often differ in size and morphology. Some clones grow quickly while others grow very slowly.
165. Continue checking cultures daily under microscope. Passage the culture as described in Section 3.3 at ratios indicated in Table 2 when first clones are emerging.
  **TROUBLESHOOTING** If wells appear empty after splitting and cultures are not able to recover and repopulate the well again, it is possible that the splitting ratio was too high (Table 2). The culture should thus be split at a lower ratio next time. If the culture has been split too harshly and cannot recover, the remainders can be split again at a lower ratio. Keep the remainders of the cultures during critical steps, i.e., when first clones emerge, because sometimes it can be difficult to estimate the growth of a culture. If unsure how to split a culture, it can be split into two wells at different ratios (e.g., 1:3 and 1:4, or 1:5 and 1:10).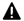 **CRITICAL STEP** Perform the Nutlin-3a counter-screen once the culture shows a clonal population of homogenous appearance and has resumed growth. Generally, wait until the culture is able to repopulate a 6-well dish within six days after being split at least 1:3. Most cultures can be split at much higher ratios (Table 2). Again, each culture should be evaluated separately and split at a too low rather than a too high ratio. First clones will emerge as early as 25 days post-treatment (D32+), while some will take up until 83 days post-treatment (D90) of the assay to be ready for screening.
166. Prepare two wells on a 6-well plate for each culture that is ready for the Nutlin-3a counter-screen. Label the plates as shown in Figure 6 (Day 1) and add 2 mL growth medium to each well.
167. Passage the culture as described in Section 3.3 to two wells of the prepared 6-well plate. Choose the splitting ratio based on the last passages so it can repopulate the well in six days.
168. The next day prepare the Nutlin-3a treatment solution in a 15 mL tube.
169. Label the plate as shown in Figure 6 (Day 2). Aspirate the medium of both culture wells. Add growth medium to the control well (-N) and the Nutlin-3a treatment solution to the other well (+N).
170. Place the plate back into the incubator and visually inspect the culture under the microscope after five days to determine the response of the culture towards Nutlin-3a (Figure 6; Day 7).
  **TROUBLESHOOTING** If the control well is not full after 5 days of treatment during the Nutlin-3a counter-screen, it is possible that the splitting ratio was too high. Passage the culture once more and rescreen at a lower splitting ratio. If the outcome of the Nutlin-3a counter-screen is unclear after 5 days of treatment, it is possible that the splitting ratio was too low or it may be a mixed-response culture. Sometimes WT-*TP53* cells can appear insensitive to Nutlin-3a when they are very full at the beginning of the treatment. Passage the culture once more, then rescreen. If still unsure about the outcome, expand the Nutlin-3a-treated and untreated well and sequence for *TP53* mutations.

#### 3.5.5. Expansion into Immortalised HUF Cell Lines—Time for Completion: ~2 Months (D32–90)

171. **NOTE** Expand all cultures with a resistant and mixed response towards Nutlin-3a. Also keep the respective Nutlin-3a treated culture. Sometimes with mixed cultures the Nutlin-3a-treated well will have to recover for ~3 days before it can be moved to a T25 flask. Also expand at least one WT culture to prepare western blot lysates from and include on the western blot gel.
172. At the end of the Nutlin-3a counter-screen label two T25 flasks with culture ID, passage number and date (e.g., 124/124N, P10, 5/10/18) and add 5 mL growth medium per flask.
173. Passage both the control (-N) and the Nutlin-3a-treated (+N) well at an appropriate ratio from the 6-well plate to the flask. Base the splitting ratio on the ratio used for the Nutlin-3a counter-screen or adjust if well was over-/under-populated at the end of the Nutlin-3a counter-screen.
  **NOTE** Take the different surface areas into account.
174. Once the T25 flask is full repeat step 173 but passage the cells into a T75 flask with 12 mL of growth medium.
175. Once the T75 flask is full, label one T75 and two T25 flasks and add fresh growth medium into each flask. Passage the culture to the new flasks.
176. When the next T75 flask is full, trypsinise the cells and use the cell solution to prepare a pellet and two frozen stocks (continue to step 178—Section 3.6).
177. Make lysates for western blotting from the two T25 flasks (continue to step 188—Section 3.7).

### 3.6. Cryopreservation of Immortalised HUF Cell Lines and Preparation of Cell Pellets for DNA Isolation—Time for Completion: 30 Min

178. Following from step 176 (Section 3.5.5), prepare frozen stocks and pellet once the T75 flask is full.
179. For each T75 flask label two 15-mL tubes with the culture ID and “Tube 1” (for pellet) and “Tube 2” (for stock).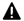 **CRITICAL STEP** It is possible to prepare frozen stocks from up to four cultures at a time. Because the freezing medium contains a high percentage of DMSO it is important that cells are moved to −80 °C immediately to avoid any cellular damage.
180. Label two cryogenic vials with culture ID, date, passage and size of flask (e.g., X-124, 10/07/18, P15, ¼ T75).
181. Label one microcentrifuge tube with culture ID and date.
182. Add half of the cell solution from step 176 (Section 3.5.5) to each of the 15-mL tubes from step 179 and centrifuge at 320× *g* for 5 min at room temperature.
183. Prepare freezing medium.
184. For pellet: transfer pellet from “Tube 1” to a microcentrifuge tube with 600 μL PBS, mix and centrifuge at 2300× *g* for 3 min at room temperature.
185. Aspirate the PBS from the pellet and store at −20 °C until DNA isolation (continue step 199—Section 3.9).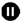 **PAUSE STEP** Cell pellets can be stored at −20 °C for several months until DNA isolation.
186. For frozen stock: Aspirate the supernatant from “Tube 2” and re-suspend the pellet in 2 mL freezing medium (for 2× 1 mL stocks) by gently pipetting up and down.
187. Add 1 mL to each of the prepared cryogenic vials and place in a Styrofoam box at −80 °C. Transfer vials to liquid nitrogen tank within 1 week.

### 3.7. Preparation of Cell Lysates for Western Blotting to Assess p53 Activation in Immortalised Clones Following Nutlin-3a Treatment—Time for Completion: 15 Min for Treatment Followed by 24 h Incubation, 15 Min for Collection of Cell Lysates

188. Once the T25 flasks seeded in step 173 (Section 3.5.5) have reached ~50% confluency prepare DMSO control and Nutlin-3a treatment solution (Section 5).
189. Label the flask with +DMSO or +Nutlin-3a and aspirate the medium. Add DMSO or Nutlin-3a treatment solution to respective flask and place flask back into incubator.
190. After 24 h label two microcentrifuge tubes with culture ID −/+N.
191. Prepare required amount of lysis buffer with protease inhibitors.
192. Wash cells once with PBS and add 250 μL lysis buffer.
193. Rotate the flask to ensure the lysis buffer coats the whole surface and collect the lysate in the corner of the flask using a cell scraper.
194. Pipette lysates into labelled microcentrifuge tubes from step 190 and store lysates at −20 °C.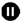 **PAUSE STEP** Cell lysates can be stored at −20 °C for several months until analysis.

### 3.8. Expression and Activation of p53 in Immortal HUFs Following Treatment with Nutlin-3a—Time for Completion: 3 Days

195. Following from step 194—Section 3.7 normalise samples as described in Section 3.4.2.
196. Follow western blot protocol as described in Section 3.4.2. Dilutions for primary and secondary antibodies can be found in Table 5 and Table 6 (Section 5).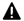 **CRITICAL STEP** When comparing Nutlin-treated and untreated samples they should be loaded next to each other. Include one *TP53*-WT culture on each gel. Load a minimum of 10 µg protein.
197. Additionally, after probing for p53, the upper membrane should be re-probed for Mdm2.
198. Follow steps 117–130.

### 3.9. DNA Isolation for TP53 Sequencing—Time for Completion: 30–90 Min Hands on Time (Depending on Sample Number), Followed by Overnight Wait

199. Using pellets prepared in Section 3.6, isolate the DNA using the Gentra Puregene Cell Kit B DNA isolation according to manufacturer’s instructions.
200. The next day quantify concentration and purity of the DNA by measuring UV absorbance at 230, 260 and 280 nm using a NanoDrop microvolume spectrophotometer.
201. Dilute DNA samples to 100 ng/µL with DNA hydration solution.
202. Store DNA at −20 °C until further use.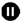 **PAUSE STEP** DNA can be stored at −20 °C for several months before PCR analysis.

### 3.10. PCR Amplification of Exons 4–9 of TP53 from HUF Clone DNA and Sample Preparation for Sequencing—Time for Completion: Half Day

203. Switch on UV light in PCR hood for ~20 min.
204. Prepare a Styrofoam box with ice.
205. Prepare or thaw PCR reagents [primers: 4F, 4R, 5F, 6R, 7F, 7R, 8F, 9R (Table 7—Section 5), REDTaq^®^ ReadyMix™] and DNA samples (see step 202—Section 3.9).
206. Prepare a layout for a 96-well plate or label 0.5-mL tubes including a negative water control. Four tubes will be required per sample analysing exon 4, exons 5_6, exon 7 and exons 8_9, respectively.
207. Add 23 µL Master Mix to each tube/well (Table 8—Section 5).
208. Add 2 µL DNA to each tube/well. Pipette up and down and make sure to change the tip after each tube/well.
209. Place samples in thermal cycler and run programme shown in Table A1 (Appendix A).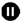 **PAUSE STEP** Samples can be stored at 4 °C until the next day or for a few days at −20 °C before continuing with the next part of the protocol.
210. Prepare agarose gel cast by taping openings with autoclave tape.
211. Weigh 4 g of ultra-pure agarose in a 200-mL beaker and add 200 mL 1X TBE buffer (2% agarose).
212. Heat agarose in microwave by boiling for ~3 min until dissolved.
  **NOTE** Microwave ovens vary. Start with 1 min, take the solution out and mix it by swirling gently. Repeat until solution is clear. Be very careful when taking the solution out of the microwave. Wear appropriate protective clothing (lab coat and goggles).
213. Pipette 10 μL ethidium bromide (10 µg/µL) into molten agarose (final ethidium bromide concentration: 0.5 µg/µL).
  **NOTE** Ethidium bromide is mutagenic. Wear appropriate clothing and handle with care.
214. Pour agarose slowly in gel cast making sure not to create bubbles and add combs to gel cast.
215. Allow the gel to set for ~40 min.
216. Take out comb, remove autoclave tape and place gel cast in gel tank.
217. Fill the gel tank with approximately 1 L 1X TBE making sure the electrodes and gel are covered.
218. Pipette 6 µL DNA ladder into the first well. Pipette 2 µL per well of each PCR reaction onto the gel.
219. Close lid of gel tank and attach cables accordingly. Run gel at 140 V for ~45 min.
220. Visualise bands by exposure to UV light and save image. The expected product sizes are shown in Table 7 (Section 5).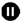 **PAUSE STEP** Amplified products can be stored at 4 °C until the next day or for a few days at −20 °C before continuing to next section.
221. Clean-up the PCR products from step 209 using the peqGOLD Cycle-Pure Kit according to the manufacturer’s instructions.

### 3.11. TP53 Sequencing and Analysis—Time for Completion: 2 Days

**Figure 7:**
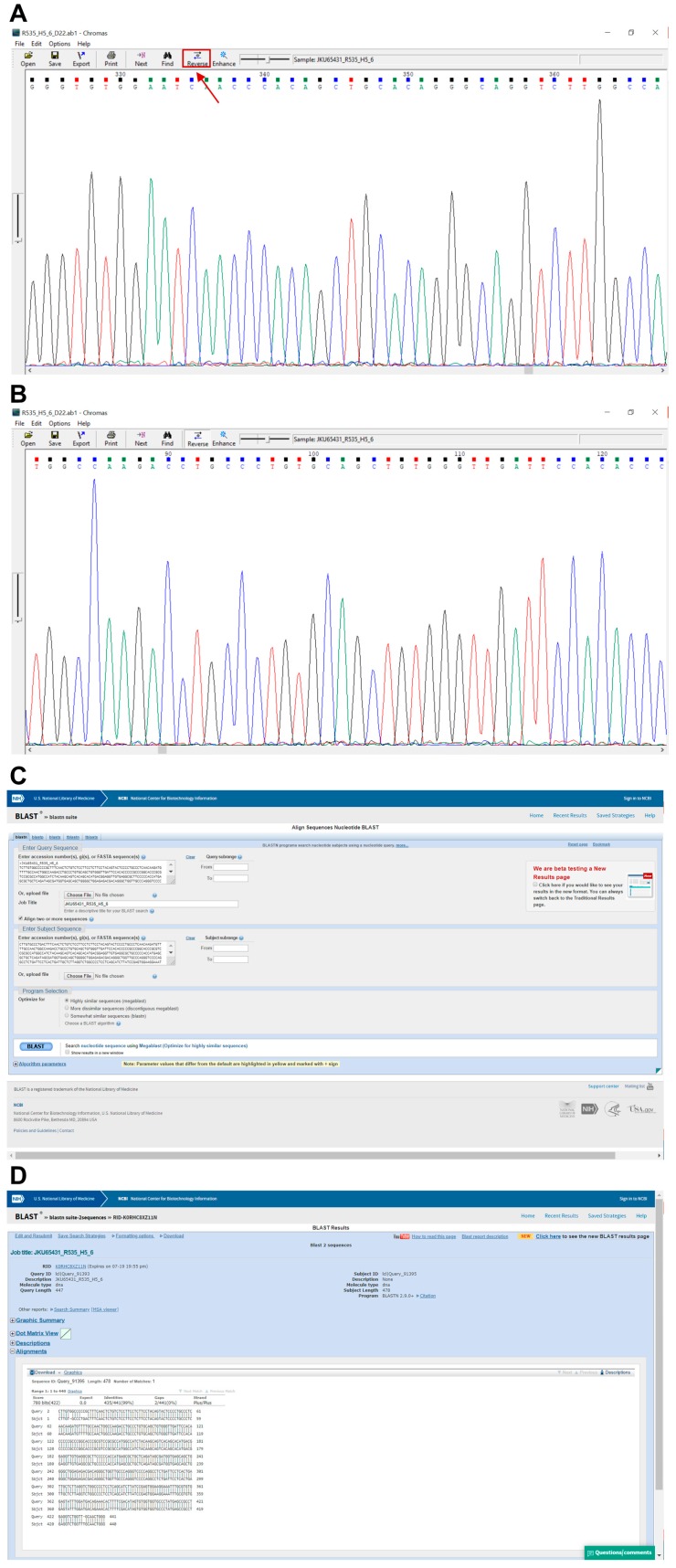
*TP53* sequence analysis using Chromas software and BLASTN sequence alignment. (**A**) Chromatogram as shown with the software Chromas. The red arrow indicates the ‘reverse’ button. (**B**) Chromatogram as shown by Chromas after being reversed. (**C**) BLASTN website showing where to paste the FASTA sequence of the sample (query) and the reference *TP53* sequence (subject). (**D**) Alignment of both sequences as shown by BLASTN.

**Figure 8:**
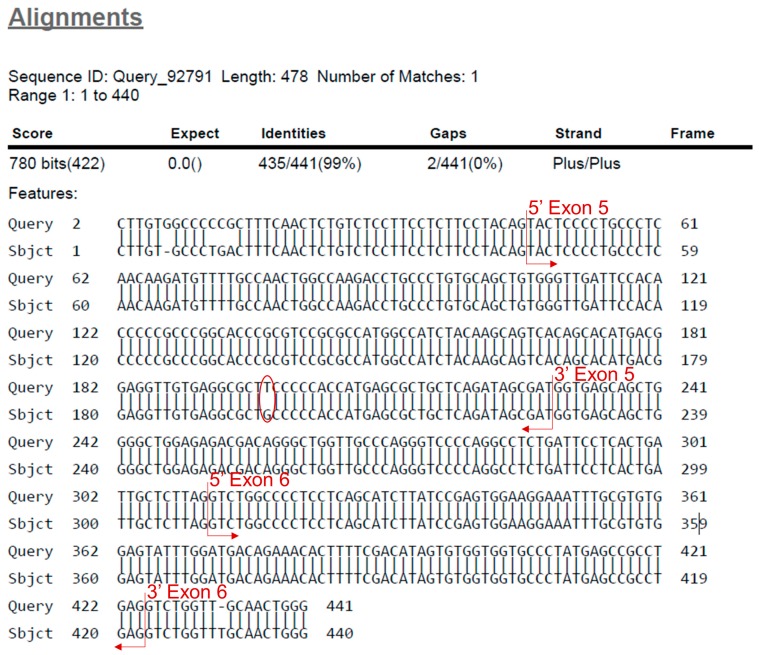
Identifying *TP53* mutations. Representative image of a *TP53*-mutant harbouring a G:C to T:A mutation (red circle) in exon 5. 3′ and 5′ ends are shown in red.

**Figure 9:**
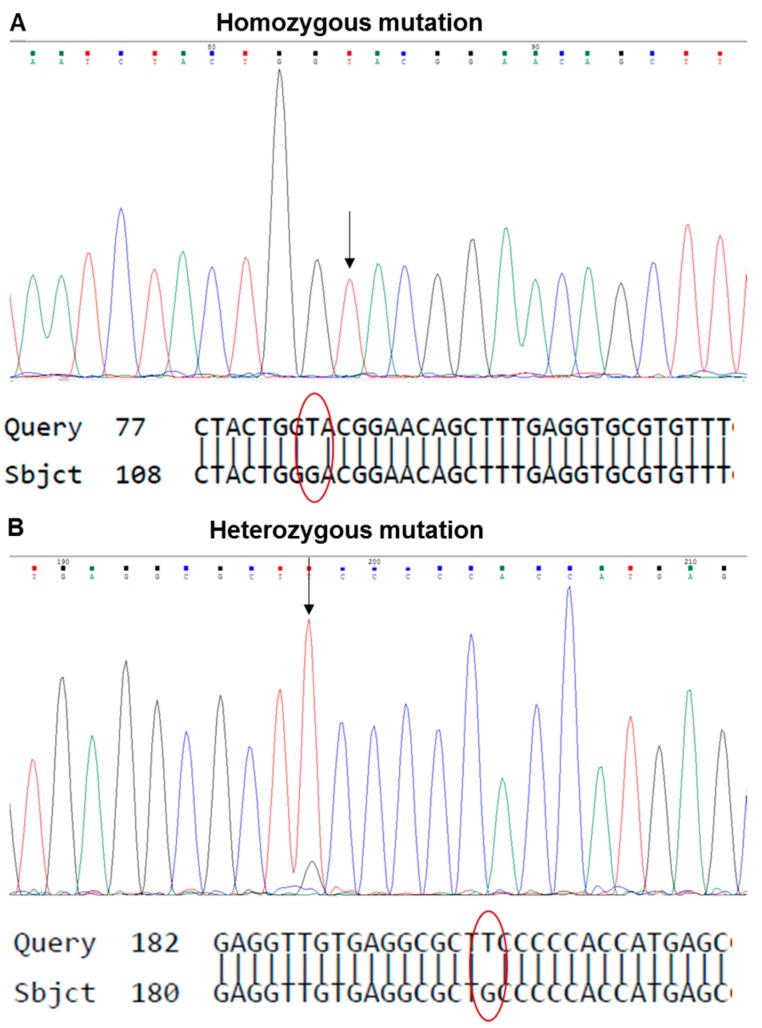
Example of sequencing chromatograms and aligned sequences. Shown are chromatograms and the sequence of *TP53*-mutant cultures both containing G:C to T:A transversions. Using the chromatogram, the mutations were identified as (**A**) homo-hemizygous and (**B**) heterozygous. The red circle indicates the mutation in the aligned test sequence (query) and the reference sequence (subject).

222. Perform Sanger dideoxy sequencing on each product from step 221 (Section 3.10) with sequencing primers shown in Table 9 (Section 5). Sanger sequencing is offered by various DNA service providers such as GENEWIZ (www.genewiz.com).
223. Once the sequencing has been performed you will be provided with an .ab1 file.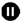 **PAUSE STEP** The analysis of the .ab1 files can be performed at any preferred time.
224. Open the .ab1 file with a suitable software (e.g., Chromas, Technelysium Pty Ltd., Australia) and examine the chromatogram visually (Figure 7A,B).
225. Reverse and complement the sequence by pressing “reverse” for all exons except exon 8_9 (Figure 7A).
226. Export the FASTA sequence of each exon using “Ctrl+B”.
227. Open the Basic Local Alignment Search Tool for Nucleotides (BLASTN) from the National Center for Biotechnology Information (NCBI) (https://blast.ncbi.nlm.nih.gov/Blast.cgi).
228. Paste the FASTA sequence from step 226 into “Query Sequence” box (Figure 7C).
229. Copy and paste the *TP53*-WT sequence from Table A2 (Appendix A) into the “Subject Sequence” box (Figure 7C).
230. Click “Blast” and print the result (Figure 7C).
231. Find the beginning and end of the exon. Identify the mutation by looking for missing bases, mismatched bases or insertions (Figure 8).
232. Check the mutation in the chromatogram visually to identify whether it is a homo-/hemi- or heterozygous mutation (Figure 9).
233. Use the mutation feature search tool of the IARC TP53 mutation database (http://p53.iarc.fr/TP53GeneVariations.aspx) to assess information about the specific mutation.
234. Continue analysis with the other clones.

## 4. Expected Results

### 4.1. Optimisation of Carcinogen Treatment Conditions for HIMA

**Figure 10:**
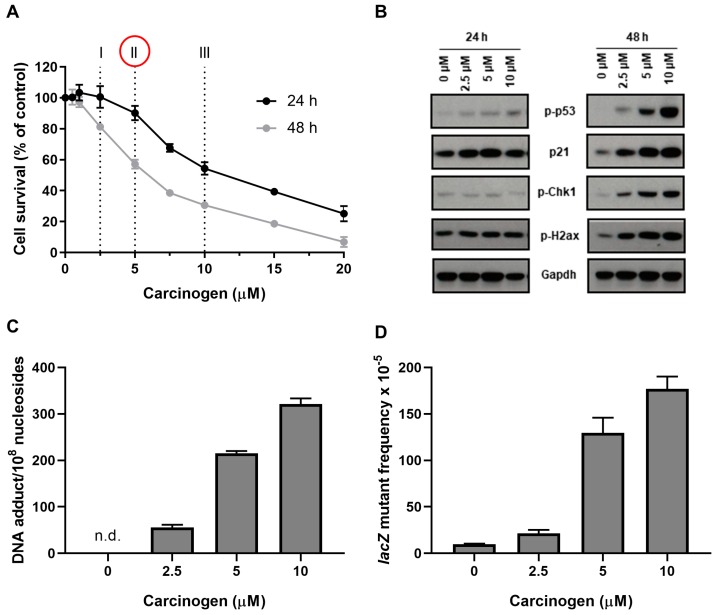
Optimisation of treatment conditions for the HIMA (simulated experiment). (**A**) Cell survival of primary HUFs after carcinogen treatment for 24 and 48 h. Cells treated with solvent served as control. Values represent mean ± SD (n = 3). Carcinogen concentrations I (~60–80% viable cells), II (~40–60% viable cells) and III (~20–40% viable cells) were used for testing DDR, DNA adduct formation and *lacZ* mutagenicity. (**B**) Expression of DDR proteins (i.e., p-p53, p21, pChk1, and pH2ax) by western blotting in primary HUFs after carcinogen treatment for 24 and 48 h. Cells treated with solvent served as control. Representative images of the western blotting are shown, and at least duplicate analysis was performed from independent experiments. Gapdh expression was used a loading control. (**C**) DNA adduct formation in primary HUFs after carcinogen treatment for 48 h. Cells treated with solvent served as control. Values represent mean ± SD (n = 4). n.d. = not detected. (**D**) Mutant frequency at the *lacZ* locus in primary HUFs after carcinogen treatment for 48 h. HUFs were treated with the indicated concentrations and then allowed to proliferate for 4 days to fix DNA damage into mutations. Cells treated with solvent served as control. *LacZ* mutant frequencies were calculated as the number of mutant colonies per number of recovered transformants. Values represent mean ± SD (n = 5). NOTE: Based on all assays conducted, we would recommend selecting carcinogen concentration II for the HIMA using a treatment period of 48 h (see red circle in panel A).

Because the HIMA is a long and labour intensive assay, experimental treatment conditions should be optimised prior to initiating the HIMA. Agents of interest should be tested for their ability to cause DNA damage in HUFs, and conditions must be optimised (i.e., concentration and/or exposure time) to ensure that the level of damage is high enough to potentially induce mutations while most cells remain viable. Existing knowledge of the mechanism(s) of action of the tested carcinogen can be a guide to choosing the experimental treatment conditions to test. When assessing cytotoxicity, the first test of concentration should be based on concentrations used in other cell assays reported in the literature. Initially, it is recommended to determine cell survival at a variety of concentrations and time-points (usually up to 48 h). Typically, for experiments in a 96-well plate, as described in this protocol, eight concentrations of the carcinogen and the solvent control can be examined in six replicates (Figure 10A). Many carcinogenic agents require metabolic activation into reactive intermediates to exert their DNA damaging effects [30] and thus may require a longer treatment period (up to 48 h). When testing reactive intermediates shorter treatment periods (6–24 h) should be considered. To ensure that cells remain viable after initial treatment (i.e., continue to divide, fix *TP53* mutation and continue to proliferate) it can be helpful to assess cytotoxicity after an additional 24 h sampling period. Our laboratory commonly uses the crystal violet staining assay as it is inexpensive, quick and reliable. We have also used resazurin-based assays (e.g., Deep Blue Cell Viability, Biolegend) but have found that some cytotoxic carcinogens, such as BaP, can stimulate the metabolism of resazurin even while cells are dying, giving incorrect viability results (unpublished observation).

To counteract the potentially deleterious effects of DNA damage, cells have evolved multifaceted mechanisms—collectively termed DDR—to detect DNA lesions, signal their presence and promote DNA repair [31]. Evaluating the induction of DDR signalling markers can help to estimate the presence of DNA damage in HUFs. However, it is important to note that the induction of DDR is not necessarily indicative of the mutagenic potential of the agent studied [21]. To standardise the experimental approach when assessing the expression of DDR markers, concentrations that cause 60–80%, 40–60%, and 20–40% cytotoxicity are typically selected. Usually, we examine the induction of phosphorylation or expression of four DDR proteins: phospho-p53, p21, phospho-Chk1 and phospho-γ-H2ax (Figure 10B). Only those concentrations that show a clear induction of several DDR proteins should be considered for the HIMA.

Initial DNA damage, persistence of the damage, as well as the mutagenic specificity of individual DNA adducts, all contribute to the mutagenic potency of the carcinogens tested [26]. For many mutagens, DNA adduct formation correlates well with mutation frequency in standard mutation bioassays [32,33]. Thus, not only can the formation of premutagenic DNA adducts be used as a measure of the ability of the HUFs to metabolically activate the test agent, but also the extent of DNA binding (i.e., DNA adduct levels) helps to demonstrate that under the selected experimental conditions (i.e., concentration tested and exposure time) sufficient DNA damage is present to induce mutagenicity (Figure 10C). Usually the same concentrations as those selected for the assessment of DDR markers are tested for DNA adduct formation. Only those concentrations that show detectable levels of DNA adducts should be considered for the HIMA. Several methods (e.g., ^32^P-postlabelling, mass spectrometry) are available to detect and quantify DNA adducts [26]. All methods available for DNA adduct detection have their strengths and weaknesses, so the method of choice has to be decided on a case-by-case basis considering the carcinogen to be studied, prior knowledge of the carcinogen-induced DNA adducts formed and the infrastructure available in the host laboratory [26]. Alternative approaches to assess DNA damage (e.g., comet assay) have been considered in HUFs previously [34]. Again, only those concentrations that show a clear induction of DNA damage should be considered for the HIMA.

Bulky DNA adducts can be removed by DNA repair (e.g., nucleotide excision repair (NER)) [35]. As described earlier, we developed a Hupki mouse strain harbouring a knockout allele for a critical NER component, Xpa (xeroderma pigmentosum complementation group A) [15]. HUFs deficient in Xpa are incapable of removing bulky DNA adducts from their genomes. Thus, utilising Xpa^−/−^ HUFs instead of Xpa^+/+^ HUFs may increase the *TP53* mutation frequency in the HIMA. However, as reported previously, the mutation frequency was not increased in Xpa^−/−^ HUFs although an increased number of mutations was found on the transcribed strand in Xpa^−/−^ HUFs [15].

As the HIMA takes several months to complete, using a short-term reporter gene mutation assay (in a manner that is not dependent on the selection within HUFs) is another approach to optimise experimental treatment conditions prior to initiation of the HIMA. Mutagenicity can be rapidly screened in HUFs using the *lacZ* system [29]. We previously created HUFs in which the pUR288 plasmid locus has also been integrated [13]. The pUR288 plasmid can be extracted from the genomic DNA of mutagen-treated HUFs following 1–3 days of proliferation and *lacZ* mutations identified by selection in *E. coli* host cells [29]. Usually the same concentrations as those selected for the assessment of DNA adduct formation are tested for *lacZ* mutagenicity (Figure 10D). Only those concentrations that show measurable levels of mutagenicity above background should be considered for the HIMA. It is also noteworthy that using the *lacZ* reporter gene in MEFs it has been shown that growing MEFs at standard oxygen (i.e., 20%) conditions increases the *lacZ* mutant frequency threefold compared to MEFs cultured at 3% oxygen. These mutations were mostly G→T transversions, which is the signature mutation for 8-oxo-guanine and an indicator for oxidative damage to DNA. This mutation type was not observed in cultures kept at low oxygen [7], highlighting the importance of performing carcinogen treatment in the primary HUF at 3% oxygen as recommended in this protocol.

### 4.2. Cell Morphology during HIMA

**Figure 11:**
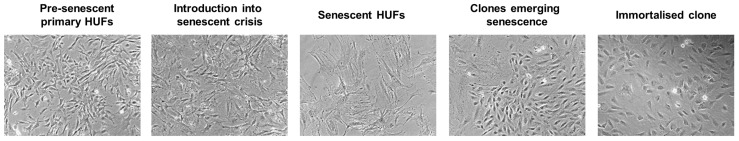
Morphology of HUFs at different stages of the HIMA. Spindle-shaped primary HUFs become enlarged and flattened during senescence, while immortalised clones develop with various morphologies. Photomicrographs of cells growing in adherent monolayers were taken at ×100 magnification.

During the different stages of the HIMA the morphology of the cells will change (Figure 11). While primary HUFs used at the beginning of the HIMA are characterised by a spindle shape, the cells during the senescence crisis are flattened and more elongated. The first immortalised clones can emerge as soon as 25 days post-treatment (D32+). Immortalised clones appear in various sizes and morphologies and grow at different rates. They can be evenly spread across the well, but some of them grow in patches.

### 4.3. Results of the Nutlin-3a Counter-Screen

**Figure 12:**
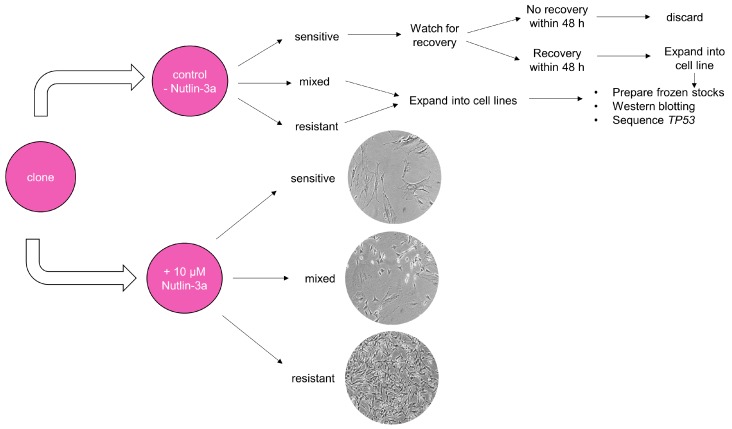
Schematic representation of the Nutlin-3a counter-screen and possible outcomes. Once clones emerge from senescent crisis, the Nutlin-3a counter-screen is performed. Cells are treated for five days with 10 µM Nutlin-3a to determine the response of the immortalised HUF. Mixed and resistant (mutant-*TP53*) cultures are expanded into immortalised cell lines. Sensitive (WT-*TP53*) cultures are watched for recovery and discarded if they do not recover within 48 h.

Once a culture contains a fully clonal population the Nutlin-3a counter-screen is performed. Possible outcomes of the screen are illustrated in the scheme shown in Figure 12.

**Figure 13:**
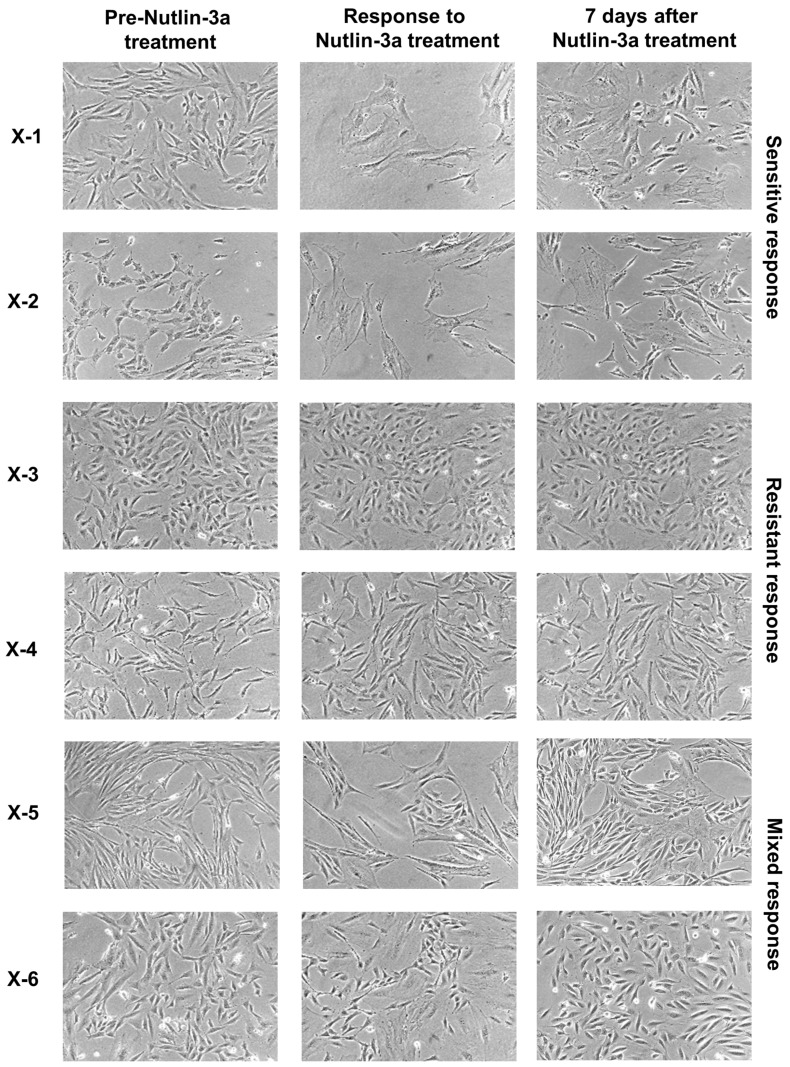
Morphology of various outcomes of the Nutlin-3a counter-screen. X-1 to X-6 shows responses of different HUF clones. Photomicrographs of cells growing in adherent monolayers were taken at ×100 magnification.

After a 5-day treatment with 10 µM Nutlin-3a the morphology of the Nutlin-3a-treated culture is compared with the untreated culture and the response to Nutlin-3a is assessed (see Figure 13). A culture can respond in a sensitive manner, which generally indicates a *TP53*-WT status, or in a mixed/resistant way, which indicates *TP53*-mutant status. Nutlin-3a-sensitive cultures show enlarged and significantly flattened cells that look very similar to senescent HUFs cultured at atmospheric oxygen. The growth of the sensitive cultures is inhibited after Nutlin-3a treatment, i.e., the well treated with Nutlin-3a is not full after 5 days. It is very common for a sensitive culture to fully recover after 3–7 days. Additionally, very rarely some sensitive clones carry *TP53* mutations [22]. Resistant cultures show the same morphology and a similar growth rate to untreated cultures. Resistant cultures can fully populate the well within 5 days. Mixed cultures show a mix of senescent, sensitive cells together with areas of the well where the resistant cells are growing. A mixed-response culture will not be full after the 5-day Nutlin-3a treatment.

**Figure 14:**
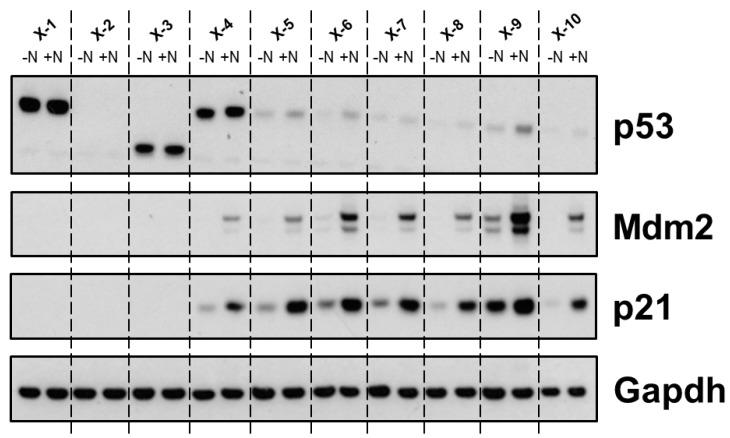
Western blot analysis of immortalised HUFs after treatment with (+N) or without (-N) 10 μM Nutlin-3a. Blots show the response of various *TP53*-mutant (X-1–5) and *TP53*-WT (X-6–10) clones. Gapdh was used as a loading control.

To further investigate the impact of the respective *TP53* mutation on the p53 signalling pathway expression and induction of p53 pathway proteins (e.g., p21, p53 and Mdm2) are analysed by western blotting. For this immortalised HUFs are treated with Nutlin-3a for 24 h. A typical response of five *TP53*-mutant *TP53*-WT clones is shown in Figure 14. While treatment with Nutlin-3a leads to the induction of p53 pathway proteins (p53, p21, Mdm2) in *TP53*-WT clones (X-6–10), the response in *TP53*-mutants can differ (Figure 14). Most mutants exhibit very strong basal p53 expression, which is not induced upon treatment with Nutlin-3a (X-1–2), while some mutations impact the size of p53 (X-3). Typically, most *TP53*-mutants do not show induction of p53 pathway members p21 or Mdm2 after Nutlin-3a treatment, but some mutants retain this ability (X-4 and X-5). Additionally, apoptosis and senescence signalling in HUF clones has been investigated by others using western blotting, but this is usually not part of the standard *TP53* mutation assay in HUFs [14,36].

### 4.4. Results of TP53 Mutation Analysis in HUFs

**Figure 15:**
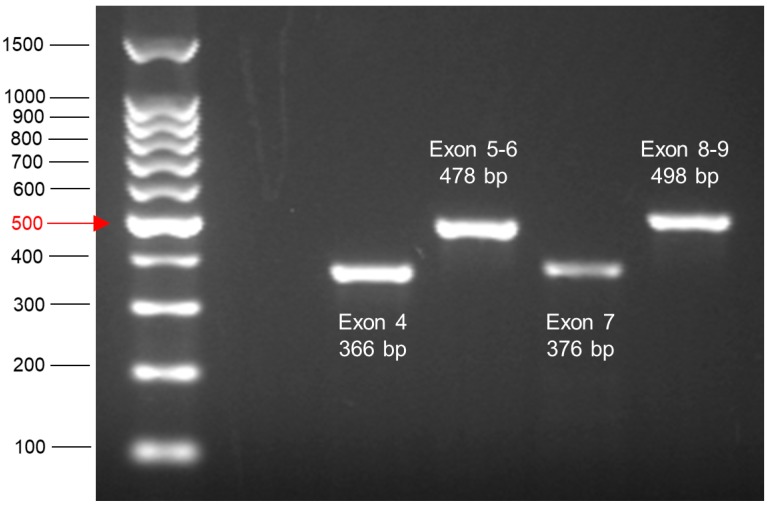
*TP53* products on 2% agarose gel. The intensity of the 500 bp DNA marker band (red) is equivalent to 100 ng DNA, while the intensity of the other bands (black) is equivalent to 50 ng DNA. By comparing the intensity of the PCR products with the bands of the DNA ladder it is possible to estimate the amount of product present in the sample.

After amplifying exons 4–9 by PCR, an agarose gel should be run to ensure that the PCR was successful and that the amount of DNA is enough for Sanger sequencing. A DNA ladder is run alongside the samples, which helps to estimate the sizes of the PCR products and the DNA amount. The size and intensity of each PCR product is compared to the ladder, where the 500 bp band is equivalent to 100 ng DNA and the other bands are equivalent to 50 ng DNA. An example of a representative agarose gel is shown in Figure 15.

Once the sequencing data are available and FASTA sequences have been analysed by alignment against the human *TP53* reference sequence, variations (e.g., single base substitutions, deletions) are assessed using the mutation validation tool of the IARC TP53 mutation database. *TP53* mutations can be classified as either homo-/hemizygous or heterozygous. Typical results and a way to display them are illustrated in Table 10. Features that should be listed in the table are: codon number where the mutation is located, mutation type, strand on which the mutation is harboured, WT- and MUT-codon bases, zygosity, coding change, and the functional activity as assessed by Kato et al. [37] in a yeast promotor transactivation assay. Based on this yeast assay the impact of the mutation can be categorised as functional, non-functional or partially functional.

### 4.5. Carcinogen-Induced TP53 Mutations in HUFs

**Figure 16:**
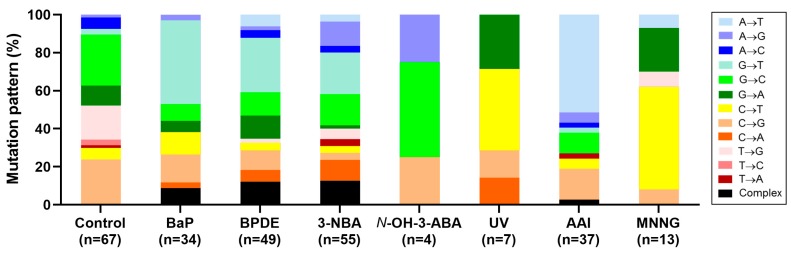
Comparison of the different mutation patterns found in *TP53* in spontaneously immortalised HUFs (includes data from [14]) and in those treated with BaP, BPDE (data from Xpa^−/−^ and Xpa^+/+^ HUFs combined), 3-NBA (data from Xpa^−/−^ and Xpa^+/+^ HUFs combined), *N*-OH-3-ABA, UV light, AAI or MNNG. For experimental conditions and abbreviations refer to Table 11. n = number of *TP53* mutant clones generated for each agent.

Environmental carcinogens that have been examined using the HIMA are summarised in Table 11. In each case, a unique *TP53* mutation pattern was generated in the HIMA, which differed from that found in control HUFs that had undergone spontaneous immortalisation (Figure 16).

Depending on the carcinogen tested the frequency of *TP53*-mutants was 11–33% while untreated controls had a *TP53* mutant frequency of 4–13%. Agents studied include BaP, which is often used as a model for polycyclic aromatic hydrocarbons (PAH) that are formed during the incomplete combustion of organic material and are ubiquitous in the environment [33,41]. After metabolic activation by cytochrome P450 enzymes BaP is converted into its reactive intermediate BPDE, which forms pre-mutagenic adducts with DNA (i.e., 10-(deoxyguanosin-*N*^2^-yl)-7,8,9-trihydroxy-7,8,9,10-tetrahydro-BaP or dG-*N*^2^-BPDE) [15,42]. Using the HIMA the *TP53* mutant frequency after BaP treatment (1 µM for 4–6 days) was 23% [11] and the *TP53* mutant frequency for BPDE (0.5 µM for 2 h) was 16–23% [15]. However, it is important to note that the *TP53* mutant frequency in the spontaneously immortalised HUF clones differed considerable between the two studies. Whereas the *TP53* mutant frequency after BaP treatment was only 1.8-fold higher than background, for BPDE the mutation frequency was 4.2-fold higher [11,15]. The predominant mutation type in both studies was G→T transversion, which is in line with the formation of pre-mutagenic dG-*N*^2^-BPDE adducts. 3-NBA, a nitro-PAH present in urban air pollution and diesel exhaust particles, had a *TP53* mutant frequency of 18–21% [13,38]. In the HIMA, G→T transversion was the predominant mutation type induced by 3-NBA in *TP53* which is consistent with the prevalence of 3-NBA-DNA adducts formed at guanine in DNA [13,43]. Exposure to ultraviolet light induced a *TP53* mutant frequency of 25%, with C→T transitions at dipyrimidine sites being the predominant mutation type in *TP53* [39], which is in line with the formation of DNA photoproducts (i.e., cyclobutane pyrimidine dimers and (6-4) pyrimidine-pyrimidone photoproducts) [44]. The *TP53* mutation pattern induced by the herbal drug aristolochic acid (AA) was dominated by A→T transversions which is in accordance with the formation of pre-mutagenic DNA adducts at adenine in DNA [45,46]. The *TP53* mutant frequency in AA-exposed HUFs was 21–33% [12,39,40].

### 4.6. Investigating Human Cancer Aetiology Using the HIMA

As shown in Table 11 and Figure 16 the HIMA has been used successfully to analyse the mutagenic potential and the *TP53* mutation pattern for a variety of environmental carcinogens. In several cases, the *TP53* mutation pattern generated corresponded to the pattern found in human tumours where exposure to these agents has been documented: (a) UV light, which is linked to human skin cancer, leads to the preferential induction of C→T and CC→TT mutations; (b) BaP or its reactive metabolite BPDE, which are associated with tobacco smoke-induced lung cancer, predominantly induce G→T mutations; and (c) AA, which is linked to urothelial cancer, and leads to a prevalence of A→T mutations. These data have been recorded in the IARC TP53 Database (www.p53.iarc.fr) which provides the basis to exploit the HIMA to examine current hypotheses on the endogenous or exogenous factors responsible for mutations in human cancers.

**Figure 17:**
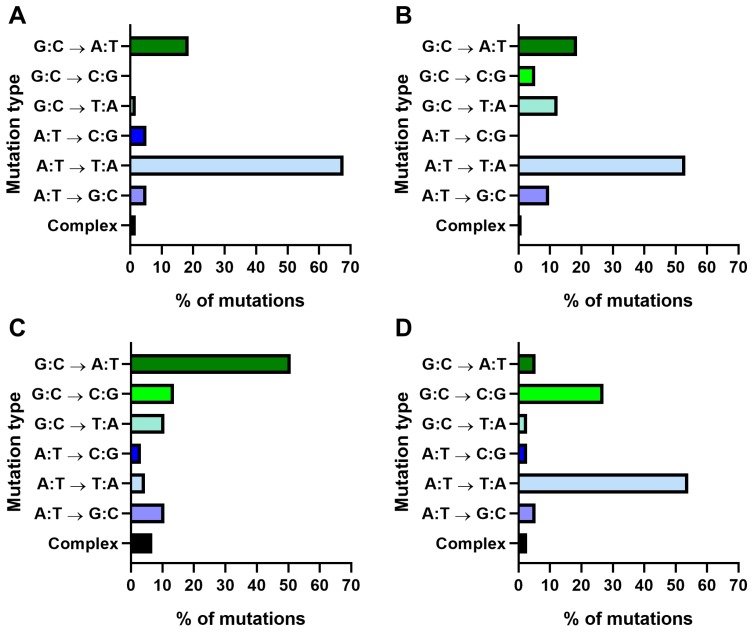
*TP53* mutation patterns found in (**A**) urothelial tumours of AA-exposed patients suffering from BEN originating from Croatia, Serbia, Bosnia, and Romania [47], (**B**) urothelial tumours of patients suffering from AAN in Taiwan [47], (**C**) urothelial tumours not associated with AA exposure [47] and (**D**) immortalised HUFs treated with AA [12,39,40].

Using AA as an example, a high prevalence of A→T mutations in *TP53* is found in urothelial tumours of AA-exposed patients suffering from Balkan endemic nephropathy (BEN) originating from Croatia, Serbia, Bosnia, and Romania [47] (Figure 17A). The same *TP53* mutation pattern (i.e., A→T mutations) is found in urothelial tumours of patients suffering from aristolochic acid nephropathy (AAN) in Taiwan [47] (Figure 17B). The A→T mutation type is otherwise rare in urothelial tumours [46] (Figure 17C). As shown in Figure 17D the *TP53* mutation pattern induced by AA in immortalised HUF cell lines closely mimics the pattern seen in AA-induced urothelial tumours in humans [12,39,40]. Therefore, the HIMA has sufficient specificity to make it applicable to other environmental mutagens that putatively play a role in cancer aetiology.

**Figure 18:**
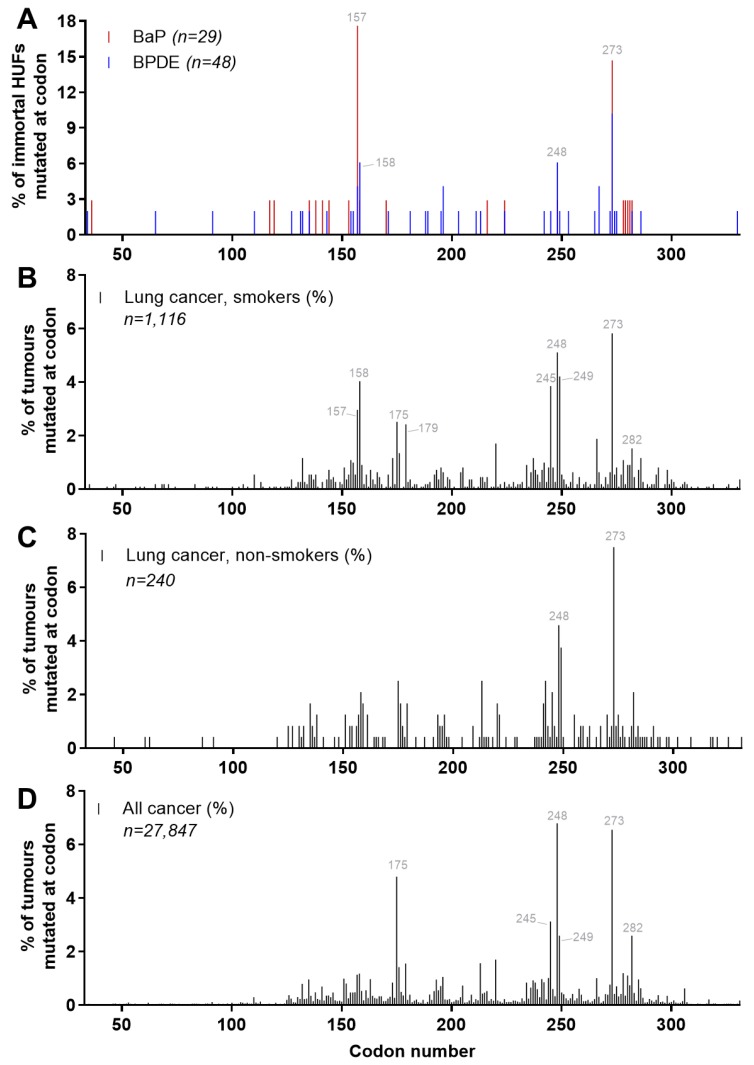
Codon distribution of *TP53* mutations (**A**) induced by BaP and BPDE in HUFs [11,15,25] and the spectrum found in lung cancer of (**B**) smokers, (**C**) non-smokers and (**D**) all cancers in exons 4–9. Reference for human cancer mutation distribution acquired from IARC TP53 mutation database, R20 July 2019 [48]. Exclusions for lung cancer mutation distribution: radon, asbestos, mustard gas, and coal [49,50]. Mutation hotspots are indicated in grey.

As has been mentioned, many environmental carcinogens induce hotspot mutations in *TP53* and these are often cancer specific [24,43]. As shown in Figure 16 and Figure 18 the predominant mutation type in immortalised HUFs treated with BaP or its reactive metabolite BPDE is G→T and induced at several mutational hotspots in the *TP53* gene [11,15,25]. The mutation pattern found in human lung cancer from smokers is also characterised by G→T transversions and commonly found at hotspot codons 157, 158, 175, 245, 248, and 273. As shown in Figure 18B, these mutational hotspots frequently observed in smokers’ lung tumours are less common in non-smokers’ lung cancer. In BaP/BPDE-treated HUFs, codons 157, 158, 248, and 273 are also recurrent sites of mutation, with a significant proportion being G→T indicating that data collected in the HIMA are consistent with the hypothesis that PAHs such as BaP have a direct role in causing *TP53* mutations in smokers’ lung tumours [24].

### 4.7. Future Perspectives: Investigating Mutational Signatures by Whole Genome Sequencing in Immortalised HUFs after Carcinogen Exposure

Several recent publications have reported using the HIMA immortalisation assay in conjunction with massively parallel sequencing (next-generation sequencing), conducted at either the whole-exome or whole-genome levels. Proof-of-principle reports have revealed successful identification of genome-wide mutational signatures of AAI, BaP, MNNG, UV light class C (UVC), as well as of the ectopically expressed activation-induced cytidine deaminase (AID). These signatures correspond reasonably well with the mutational signatures of the same exposures extracted computationally from the human cancer genomics data repositories [51,52]. Such scaled-up HIMA assay involving various chemical exposures have revealed remarkable gene-level recurrence of non-silent mutations in the mouse orthologues of known human cancer genes, accumulating across dozens of clones, including mutual exclusivity of mutations in components interacting functionally within the same protein complex. Thus, HIMA-derived cell lines can be used to study numerous candidate cancer driver events can be studied with biological relevance for functional impact on cell survival and immortalisation [53]. Lastly, the HIMA coupled with next generation sequencing and DNA adduct analysis has yielded a novel mutational signature of glycidamide, the reactive metabolite of acrylamide present in human diet and in tobacco smoke. Tailored computational analyses of the vast pan-cancer data were then able to identify the glycidamide signature in an unexpectedly large number of cancer types [54]. Collectively, these studies exemplify the future potential of the HIMA assay for systematic screening of carcinogenic compounds for robust mutagenic effects and functional impact, and they signal new in-depth insights into aetiology and development of cancer while generating important mechanistic evidence for adopting relevant preventive measures.

## 5. Reagents Setup

- Growth medium: Mix 500 mL high glucose DMEM with 50 mL fetal bovine serum and 5 mL penicillin/streptomycin solution (10,000 U/mL/10,000 µg/mL). Label the bottle with the preparation date and store at 4 °C for up to 2 weeks.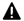 **CRITICAL STEP** All culture work must be carried out in a Class II biosafety cabinet to avoid assay culture contamination.
- Freezing medium: Prepare 1 mL freezing medium per frozen stock. Always make 1 mL extra. For example, for 2 frozen stocks prepare 3 mL by adding 300 μL DMSO into 2.7 mL growth medium.
- Carcinogen stock solution: Prepare the stock solution by dissolving an appropriate amount in DMSO or another appropriate solvent. Make aliquots which can be store for several years at −20 °C or −80 °C dependent on the agent tested.
  **NOTE** Take care when handling carcinogenic compounds. Dispose waste appropriately.
- Carcinogen treatment medium (6-well plate): Prepare 2 mL per well and include 1 mL extra. For example, if treating 120 cultures pipette 241 mL medium into a sterile flask and add the appropriate volume of carcinogen stock solution. Make sure the carcinogen stock has dissolved properly before treating the cells. For controls prepare the medium in the same way but add DMSO instead of carcinogen stock solution. Ensure that the final DMSO concentration in medium does not exceed 1% (typically 0.1–0.2% final).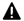 **CRITICAL STEP** Prepare the treatment medium shortly before exposing cells.
- 0.1% Crystal violet: Dissolve 500 mg of Crystal violet powder in 500 mL 10% ethanol. The solution can be stored at room temperature for up to 24 months.
- Nutlin-3a stock solution: Prepare a 10 mM stock solution by dissolving the appropriate amount of Nutlin-3a in DMSO. Make aliquots which can be stored at −20 °C for at least two years.
- Nutlin-3a treatment solution: Prepare the required volume of treatment solution by adding 10 mM Nutlin-3a stock solution to growth medium to a final concentration of 10 µM. Make 2 mL per 6-well and 5 mL per T25 flask and always include 1 mL extra. For example, for the treatment of three cultures on a 6-well plate prepare 7 mL treatment solution by combining 7 mL medium and 7 μL 10 mM Nutlin-3a stock solution in a 15-mL tube.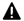 **CRITICAL STEP** Make fresh prior to use.
- DMSO treatment solution (for Nutlin-3a counter screen): Prepare the required volume of treatment solution by adding DMSO to growth medium. Make 5 mL treatment solution per T25 flask and add 1 mL extra. For example, for the treatment of three cultures on a 6-well plate prepare 7 mL treatment solution by combining 7 mL medium and 7 μL DMSO in a 15-mL tube.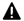 **CRITICAL STEP** Make fresh prior to use.
- Lysis buffer: Prepare lysis buffer by pipetting components shown in Table 3 into a 50-mL tube. Store at room temperature. Add protease-/phosphatase inhibitors fresh to working solution (10 μL/mL).
- Prepare albumin standards from 0–2 mg/mL as indicated in Table 4.
- Bromophenol Blue lysis buffer (BPB-LB) stock solution: Dissolve 1 mg BPB salt in 10 mL lysis buffer.
- 20X BPB/β-mercaptoethanol (bME) solution: Pipette 80 μL BPB-LB and 20 μL bME into a microcentrifuge tube and use to normalise samples. Make fresh just before use.
- MES running buffer: Add 50 mL 20X MES to 960 mL purified water. Store at room temperature for several month.
- 10X transfer buffer stock: Add 60.57 g Tris base, 288.4 g glycine and 1800 mL water into a beaker and mix on magnetic stirrer. Store at 4 °C for several months.
- 1X transfer buffer: Add 200 mL 10X transfer buffer stock to 1800 mL water and store at 4 °C.
- 10X TBS stock solution: Add 121.14 g Tris base and 175.32 g NaCl to 1800 mL water and mix on magnetic stirrer. Once everything is dissolved, adjust pH to 7.4 by adding concentrated hydrochloric acid. Complete volume accordingly and store for several months at 4 °C.
- 1X TBS with 0.1% Tween-20 (TBST): Mix 200 mL 10X TBS with 1800 mL water, then pipette 2 mL Tween-20 in. Tween-20 is highly viscous and takes some time to be removed from the tip and dissolved. Mix on magnetic stirrer. Prepare fresh on the day.
- 3% milk in TBST: Weigh 6 g powdered milk and add 200 mL TBST. Put on magnetic stirrer until dissolved. Prepare on the day.
- Primary antibody solution: Label 50 mL tubes with antibody name, date and dilution. Add volumes of 3% milk in TBST, primary antibody and sodium azide as shown in Table 5. Primary antibody solutions can be stored for several months at −20 °C.
- Secondary antibody solution: Prepare secondary antibody solution in 40 mL 3% milk in TBST as shown in Table 6 prior to use and discard afterwards.
- Primer stock solutions (100 µM): Spin primers briefly and dilute in TE-buffer to 100 µM as advised on specification sheet. Flick tube to make sure primers are dissolved. Make aliquots which can be stored for several years at −20 °C for several years.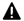 **CRITICAL STEP** Keep on ice when not in use.
- Primer working solutions (10 µM): Label eight microcentrifuge tubes with the primer name (4F, 4R, 5F, 6R, 7F, 7R, 8F, 9R) and concentration (10 µM). Pipette 180 µL nuclease-free water into each tube, add 20 µL of 100 µM primer and mix well by pipetting. Make aliquots which can be stored for several years at −20 °C for at least two thaw-freeze cycles.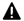 **CRITICAL STEP** Keep on ice when not in use.
- PCR Master Mix: Label four microcentrifuge tubes (4, 5_6, 7, 8_9). Calculate the required Master Mix volume as shown in Table 8 below adding 15% extra. Add components to respective microcentrifuge tubes and place back on ice until use.
- Sequencing primer working solutions (10 µM): Label four microcentrifuge tubes with the primer name (4seq, 5_6seq, 7seq, 8_9seq) and concentration (10 µM). Pipette 180 µL nuclease-free water into each tube, add 20 µL of 100 µM primer and mix well by pipetting. Make aliquots which can be stored for several years at −20 °C for several months.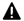 **CRITICAL STEP** Keep on ice when not in use.
- TBE buffer: Mix 1800 mL of ddH_2_O and 200 mL of 10X TBE buffer stock in a 2-L bottle. Store at room temperature and discard when precipitate forms.

## Figures and Tables

**Table 1:** Surface areas and required cell numbers and volumes for culturing primary HUFs (48 h).

Dish	T25	T75	T175	6-Well	96-Well
Surface area (cm^2^)	25	75	175	9.6	0.32
Cells/dish (× 10^6^)	0.4	1.2	2.8	0.15	0.005
Medium for culture (mL)	5	12	35	2	0.1
PBS for washing (mL)	5	10	12	2	-
Trypsin for harvesting (mL)	1	2	3	0.5	-
Medium to inactivate trypsin (mL)	4	8	17	1	-

**Table 2:** Splitting ratios for the HIMA.

Protocol Stage	Day	Passage	Ratio
Set-up	6	1	150,000 cells/well
Pre-senescence	9–10	2	1:3–1:5
	11–17	3–4	1:2.5–1:4
	19–24	5	1:1.5–1:3
Senescence	18+	5+	1:1
Clones emerging	32+	6+	1:1.5–1:2.5
Nutlin-3a counter-screen	32+	7+	1:3–1:50
Expansion into cell lines	32+	8+	1:3–1:50

**Table 3:** Preparation of lysis buffer.

	Concentration	Final Concentration	Volume (mL)
Tris pH 6.8	1000 mM	62.5 mM	1.25
EDTA pH 8.0	500 mM	1 mM	0.04
SDS	10%	2%	4
Sterile glycerol	40%	10%	5
Water	-	-	9.75
Inhibitors	100X	1X	see text

**Table 4:** Albumin standard dilutions.

ID	Albumin Concentration (mg/mL)	2 mg/mL Bovine Serum Albumin (µL)	Lysis Buffer (µL)
A	2	1000	0
B	1.5	112.5 A	37.5
C	1	150 A	150
D	0.5	150 C	150
E	0.25	150 D	150
F	0.125	150 E	150
G	0.0625	150 F	150
H	0	0	300

**Table 5:** Preparation of primary antibody solutions.

Antibody	Dilution	3% Milk in TBST (mL)	Primary Antibody (µL)	Sodium Azide (µL)
p53	1:500	10	20	100
p-p53	1:2000	10	5	100
p21	1:2000	10	5	100
pChk1	1:1000	10	10	100
pH2ax	1:1000	10	10	100
Mdm2	1:750	10	13.3	100
Gapdh	1:25,000	40	1.6	400

**Table 6:** Preparation of secondary antibody solutions.

Antibody	Rabbit/Mouse	Dilution	Secondary Antibody (µL)
p53	Mouse	1:10,000	4
p-p53	Rabbit	1:20,000	2
p21	Mouse	1:10,000	4
pChk1	Rabbit	1:10,000	4
pH2ax	Rabbit	1:10,000	4
Mdm2	Mouse	1:20,000	2
Gapdh	Mouse	1:10,000	4

**Table 7:** Primer information for PCR amplification and expected product size.

Exon	Primer Name	Forward/ Reserve	Primer Sequence (5′ to 3′)	Product Size (bp)
4	4F	Forward	GTC CTC TGA CTG CTC TTT TCA CCC ATC TAC	366
	4R	Reverse	GGG ATA CGG CCA GGC ATT GAA GTC TC	
5 & 6	5F	Forward	CTT GTG CCC TGA CTT TCA ACT CTG TCT C	478
	6R	Reverse	GCC ACT GAC AAC CAC CCT TAA CCC CTC	
7	7F	Forward	AGA TCA CGC CAC TGC ACT C	376
	7R	Reverse	CCG GAA ATG TGA TGA GAG GT	
8 & 9	8_9F	Forward	CAA GGG TGG TTG GGA GTA GA	498
	8_9R	Reverse	GTC TCT GGC ATG CGA CTC TC	

**Table 8:** Preparation of PCR Master Mix.

PCR Reaction Component	Concentration	Final Concentration	Reaction Volume (µL)
REDTaq^®^		1X	12.5
Forward primer	10 µM	20 pmol	2
Reverse primer	10 µM	20 pmol	2
DNA	~100 ng/µL	200 ng	2
Nuclease-free water	‒	‒	6.5
Total volume	‒	‒	25

**Table 9:** Primer information for Sanger sequencing.

Exon	Primer Name	Forward/Reverse	Primer Sequence (5′ to 3′)
4	4seq	Reverse	GAT ACG GCC AGG CAT TG
5 & 6	5_6seq	Reverse	GCC ACT GAC AAC CAC C
7	7seq	Reverse	CCG GAA ATG TGA TGA GAG GT
8 & 9	8_9seq	Forward	CAA GGG TGG TTG GGA GTA GA

**Table 10:** *TP53* mutations in immortalised HUF clones.

ID	Codon Number	Exon	Mutation Type	Strand ^1^	WT Codon	MUT Codon	Coding Change	Zygo- City	Activity (Kato) ^2^
X-1	91	4	G:C→A:T	NTS	TGG	TGA	W91stop	Homo-/hemi	NA
X-2	132	5	A:T→T:A	NTS	AAG	ATG	R132M	Homo-/hemi	NF
X-3	158	5	del. G	TS	CGC	_GC	Frame-shift	Homo-/hemi	NA
X-4	196	6	G:C→C:G	TS	CGA	GGA	R196G	Homo-/hemi-	PF
X-5	224	6	G:C→C:G	NTS	GAG	GAC	E224D	Hetero-	F
X-6	273	8	G:C→T:A	TS	CGT	AGT	R273S	Hetero-	NF

^1^ NTS = non-transcribed strand; TS = transcribed strand. ^2^ The overall transactivation activity of the mutant in a yeast functional assay published by Kato et al. [37]. NF = non-functional; PF = partially functional; F = functional; NA = not assessed.

**Table 11:** Summary of previous HIMAs showing the treatment conditions and *TP53* mutant frequency in carcinogen-treated and spontaneously immortalised HUF clones.

Carcinogen ^1^	Treatment Conditions	Total *TP53* Mutants	Total *TP53* Mutations Detected	Mutant Frequency (Treated) ^2^	Mutant Frequency (Untreated) ^2^	Reference
BaP #1	1 µM; 4–6 days	11	16	22.9% (11/48)	12.5% (6/48)	[11]
BaP #2	1 µM; 4–6 days	14	18	NI	NI	[25]
BPDE	0.5 µM; 2 h	16 ^4^	20 ^4^	15.7% ^4^ (16/102)	3.7% ^4^ (2/54)	[15]
		23 ^5^	29 ^5^	22.5% ^5^ (23/102)	3.7% ^5^ (2/54)	
3-NBA #1	2 µM; 5 days	19	29	21.3% (19/89)	10.4% (5/48)	[38]
3-NBA #2	1 µM; 48–96 h	11 ^4^	14 ^4^	18.3% ^4^ (11/60)	3.7% ^4^ (2/54)	[13]
		11 ^5^	12 ^5^	18.3% ^5^ (11/60)	3.7% ^5^ (2/54)	
*N*-OH-3-ABA	2 µM; 5 days	4	4	11.1% (4/36)	10.4% (5/48)	[38]
AAI #1	100 µM; 48 h	5	6	20.8% (5/24)	NI	[39]
AAI #2	50 µM; 48 h	6	6	33.3% (6/18)	NI	[12]
AAI #3	50 µM; 2/4 days	21	25	NI	NI	[40]
MNNG	20 µM; 2 h	11	13	NI	NI	[40]
UV	20 J/cm^2^ 72 h	5	7	25% (5/20)	10% (2/20)	[39]

^1^ BaP = benzo[*a*]pyrene; BPDE = benzo[*a*]pyrene-7,8-dihydrodiol-9,10-epoxide; 3-NBA = 3-nitrobenzanthrone; *N*-OH-3-ABA = *N*-hydroxy-3-aminobenzanthrone; AAI = aristolochic acid I; MNNG = *N*-methyl-*N*-nitro-*N*-nitrosoguanidine; UV = ultraviolet radiation. ^2^ Brackets show the number of *TP53* mutant clones versus the total clones analysed. ^3^ NI = not indicated. ^4^ Xpa^+/+^ HUFs. ^5^ Xpa^−/−^ HUFs.

## Appendix A

**Table A1:** PCR amplification conditions.

Step	Cycling Parameters		
	Temperature (°C)	Time (min)	Cycles
1	94	4	1
2	94	0.5	40
	60	0.5	
	72	1	
3	72	7	1
4	4–10	Hold	

**Table A2:** *TP53* reference sequences for PCR amplicons.

Exon	Sequence
4	**gtcctctgactgctcttttcacccatctac**agTCCCCCTTGCCGTCCCAAGCAATGGATGATTTGATGCTGT CCCCGGACGATATTGAACAATGGTTCACTGAAGACCCAGGTCCAGATGAAGCTCCCAG AATGCCAGAGGCTGCTCCCCCCGTGGCCCCTGCACCAGCAGCTCCTACACCGGCGGCC CCTGCACCAGCCCCCTCCTGGCCCCTGTCATCTTCTGTCCCTTCCCAGAAAACCTACCAG GGCAGCTACGGTTTCCGTCTGGGCTTCTTGCATTCTGGGACAGCCAAGTCTGTGACTTGC ACGgtcagttgccctgaggggctggcttccatgagacttcaatgcctggccgtatccc
5 & 6	**cttgtgccctgactttcaactctgtct**ccttcctcttcctacagTACTCCCCTGCCCTCAACAAGATGTTTTGCCAA CTGGCCAAGACCTGCCCTGTGCAGCTGTGGGTTGATTCCACACCCCCGCCCGGCACCCG CGTCCGCGCCATGGCCATCTACAAGCAGTCACAGCACATGACGGAGGTTGTGAGGCGC TGCCCCCACCATGAGCGCTGCTCAGATAGCGATGgtgagcagctggggctggagagacgacagggctggt tgcccagggtccccaggcctctgattcctcactgattgctcttagGTCTGGCCCCTCCTCAGCATCTTATCCGAGTG GAAGGAAATTTGCGTGTGGAGTATTTGGATGACAGAAACACTTTTCGACATAGTGTGGT GGTGCCCTATGAGCCGCCTGAGgtctggtttgcaactggggtctctgggag**gaggggttaagggtggttgtcagtggc**
7	**agatcacgccactgcactc**cagcctgggcgacagagcgagattccatctcaaaaaaaaaaaaaaaaggcctcccctgcttgccacaggtc tccccaaggcgcactggcctcatcttgggcctgtgttatctcctagGTTGGCTCTGACTGTACCACCATCCACTAC AACTACATGTGTAACAGTTCCTGCATGGGCGGCATGAACCGGAGGCCCATCCTCACCA TCATCACACTGGAAGACTCCAGgtcaggagccacttgccaccctgcacactggcctgctgtgccccagcctctgcttgcc tctgacccctgggcccacctcttaccgatttcttccatactactacccatcc**acctctcatcacatttccgg**
8 & 9	**caagggtggttgggagtaga**tggagcctggttttttaaatgggacaggtaggacctgatttccttactgcctcttgcttctcttttcctatcctg agtagTGGTAATCTACTGGGACGGAACAGCTTTGAGGTGCGTGTTTGTGCCTGTCCTGGGA GAGACCGGCGCACAGAGGAAGAGAATCTCCGCAAGAAAGGGGAGCCTCACCACGAG CTGCCCCCAGGGAGCACTAAGCGAGgtaagcaagcaggacaagaagcggtggaggagaccaagggtgcagtta tgcctcagattcacttttatcacctttccttgcctctttcctagCACTGCCCAACAACACCAGCTCCTCTCCCCAGC CAAAGAAGAAACCACTGGATGGAGAATATTTCACCCTTCAGgtactaagtcttgggacctcttatcaag tggaaagtttccagtctaacactcaaaatgccgt <human seq, mouse seq> ggtaccaaggctg**gagagtcgcatgccagagac**
